# Neuronal and glial DNA methylation and gene expression changes in early epileptogenesis

**DOI:** 10.1371/journal.pone.0226575

**Published:** 2019-12-30

**Authors:** Toni C. Berger, Magnus D. Vigeland, Hanne S. Hjorthaug, Lars Etholm, Cecilie G. Nome, Erik Taubøll, Kjell Heuser, Kaja K. Selmer

**Affiliations:** 1 Department of Neurology, Oslo University Hospital, Oslo, Norway; 2 University of Oslo, Oslo, Norway; 3 Department of Medical Genetics, Oslo University Hospital and University of Oslo, Oslo, Norway; 4 National Center for Epilepsy, Oslo University Hospital, Sandvika, Norway; 5 Department of Neurology, Section for Neurophysiology, Oslo University Hospital, Oslo, Norway; 6 Division of Clinical Neuroscience, Department of Research and Development, Oslo University Hospital, Oslo, Norway; University of Modena and Reggio Emilia, ITALY

## Abstract

**Background and aims:**

Mesial Temporal Lobe Epilepsy is characterized by progressive changes of both neurons and glia, also referred to as epileptogenesis. No curative treatment options, apart from surgery, are available. DNA methylation (DNAm) is a potential upstream mechanism in epileptogenesis and may serve as a novel therapeutic target. To our knowledge, this is the first study to investigate epilepsy-related DNAm, gene expression (GE) and their relationship, in neurons and glia.

**Methods:**

We used the intracortical kainic acid injection model to elicit status epilepticus. At 24 hours post injection, hippocampi from eight kainic acid- (KA) and eight saline-injected (SH) mice were extracted and shock frozen. Separation into neurons and glial nuclei was performed by flow cytometry. Changes in DNAm and gene expression were measured with reduced representation bisulfite sequencing (RRBS) and mRNA-sequencing (mRNAseq). Statistical analyses were performed in R with the edgeR package.

**Results:**

We observed fulminant DNAm- and GE changes in both neurons and glia at 24 hours after initiation of status epilepticus. The vast majority of these changes were specific for either neurons or glia. At several epilepsy-related genes, like *HDAC11*, *SPP1*, *GAL*, *DRD1* and *SV2C*, significant differential methylation and differential gene expression coincided.

**Conclusion:**

We found neuron- and glia-specific changes in DNAm and gene expression in early epileptogenesis. We detected single genetic loci in several epilepsy-related genes, where DNAm and GE changes coincide, worth further investigation. Further, our results may serve as an information source for neuronal and glial alterations in both DNAm and GE in early epileptogenesis.

## Introduction

Epilepsy is defined as an inherent predisposition of the brain to recurrently generate epileptic seizures [1] and affects an estimated 65 million people world-wide [2]. Temporal lobe epilepsy (TLE) is the most common subtype amongst the focal epilepsies [3], with hippocampal sclerosis being detected in 70% of drug resistant TLE patients [4, 5]. The typical clinical course of the sub-entity, mesial temporal lobe epilepsy with hippocampal sclerosis (mTLE-HS), is characterized by an initial precipitating event (e.g. cerebral trauma, inflammation, prolonged febrile seizure), a seizure-free latency period and finally the onset of spontaneous and progressive seizures [6]. This metamorphosis into a brain prone to spontaneous recurrent seizures of progressive nature, also referred to as epileptogenesis [7, 8], is characterized by a plethora of cellular and molecular changes in both neurons and glia [5, 9–18].

One third of people with epilepsy respond inadequately to treatment with the primarily symptom-alleviating antiepileptic drugs of today [19], rendering the identification of potential upstream effectors of epileptogenesis and the development of disease modifying antiepileptic drugs a task of upmost importance [20, 21].

DNAm, in the context of this paper, the methylation of CpG nucleotides in the DNA [22], plays a primordial role in brain development, cell fate, tissue specific gene expression [22–25]. It has further been shown to be modified by neuronal activity [26]. Alterations of DNAm in epileptogenesis encompass upregulation of DNA–methyl–transferases, enzymes methylating the DNA base cytosine, in human TLE patients [27], genome wide changes in DNAm during epileptogenesis [28, 29], and a later onset of spontaneous seizures in murine epilepsy models under treatment with a DNA-methyl-transferase inhibitor [30].

With the dawn of new site- and cell specific epigenetic modulators like modified CRISPR, zinc finger proteins and transcription-activator-like-effectors [31–33], the identification of potential genomic sites for antiepileptogenic intervention is of utmost importance.

DNAm in neurons and glial cells is mostly cell specific [34, 35]. Sorting of brain tissue into specific cell types prior to downstream analysis has been applied in previous studies of gene expression [36–38] and DNA methylation [39–41]. This approach provides information about the cellular origin of observed effects on the epigenome and transcriptome level and an elevated detection sensitivity of more subtle changes in DNAm and GE.

Hypotheses for this study are i) that epileptogenesis affects DNAm and GE in a cell specific manner and ii) that differential methylation (DM) in neurons and glial cells correlates with differential gene expression (DGE). To our knowledge, this is the first study to investigate DNAm and GE changes as well as their possible association in neurons and glia separately, and the first one of its kind conducted in the widely used intracortical mouse model of mTLE [15].

## Methods

### Animals

Adult male C57/BL6N mice (Janvier lab) were acquired at an age of 8 weeks, acclimatized for 4 weeks in a controlled environment (21-23° C, 12h dark/light cycles), 1–4 animals per cage, with water and food available *ad libitum*. All animal procedures were approved by the Norwegian Food Safety Authority (national ethics committee, project number FOTS: 14198) and the Centre for Comparative Medicine, Oslo University Hospital and the University of Oslo.

### Intracortical kainic acid mouse model of mTLE

We used deep cortical (juxta hippocampal) kainic acid injection to elicit an initial status epilepticus. The animal model has been described in detail in a separate paper [15]. Briefly, mice injected with kainic acid typically (91%) develop chronic epilepsy in a stagewise manner. During the acute stage directly after kainic acid injection, animals undergo a status epilepticus lasting around 4 hours (4.4 +- 2.4 hrs). This stage is followed by a clinically silent latent phase that lasts around 5 days (5+-2.9 days). The first spontaneous seizure at the end of this stage also marks the start of the last, chronic, stage of epileptogenesis, characterized by spontaneous seizures of progressive nature. For kainic acid injections, mice were anesthetized with a mixture of medetomidine (0.3 mg/kg, i.p.) and ketamine (40 mg/kg, i.p.) and kept on a heating blanket. A small craniotomy was performed in a stereotactic frame above the right hippocampus. Then kainic acid (70 nl, 20 mM, Tocris) was injected by a Hamilton pipette (Hamilton Company, NV) at a depth of 1.7 mm at the following coordinates relative to Bregma: anteroposterior −2 mm, lateral +1.5 mm (right). Anesthesia was stopped with atipamezole (300 mg/kg, i.p.). All mice received buprenorphine (0.1mg/kg, s.c.) at 4 and 12 hours after the intervention. Animals in the KA group not displaying convulsive seizures were excluded from further analysis. SH animals underwent the same procedures as described for the KA group, apart from 0,9% NaCl used instead of kainic acid for the intracortical injection.

### Tissue collection and pooling

At 24 hours after status epilepticus, cervical dislocation was performed under anesthesia and right hippocampi were extracted. After extraction, each hemisphere was placed in a 2 mL polypropylene tube, instantly shock frozen in liquid nitrogen, and stored at -80°C. Right hippocampi were pooled in 2 mL tubes from 4 (KA n = 4, S n = 4) or 2 (KA n = 4, S n = 4) mice prior to further processing. The number of mice per group (KA, SH) amounted to 8 mice per group, the number of biological samples to 3 per group (KA, SH). Tissue was kept on dry ice during pooling. See also S1 Fig.

### Fluorescent Activated Nuclear Sorting (FANS)

A modified version of a nuclear sorting protocol by Jiang *et al*. [42] was used to sort into NeuN+ (refered to as neurons) and NeuN- (refered to as glia) nuclei. Immediately after pooling, hippocampi were placed on ice, and 1 mL homogenization buffer was added to each pool. Tissue was homogenized using a GentleMACS dissociator (Miltenyi), and homogenate filtered through a 70 μm filter. A NeuN-negative control sample from adult mouse liver was processed in parallel with the hippocampal samples. Debris was removed by density gradient centrifugation, using Debris Removal Solution (Miltenyi), and nuclear pellets were resuspended in 100 μL incubation buffer per one million nuclei. Anti-NeuN Alexa Fluor488 (Merck Millipore) was added to each sample to a final concentration of 0.1 μg/mL, and samples incubated for 1 h on ice in a light protected environment. Sorting of nuclei was performed on a FACSAria (BD Biosciences). Propidium iodide (PI) was added prior to sorting, and the following strategy was used for gating (S2 Fig): 1) A nuclear gate was defined by PI-positive events. 2) Aggregated nuclei were excluded in a dot plot using the pulse width of side scatter (SSC-w) versus the pulse area of forward scatter (FSC-a). 3) NeuN-negative gate was drawn based on signal from anti-NeuN stained liver sample. NeuN-positive and NeuN-negative hippocampi nuclei were sorted into tubes, and nuclei pelleted by centrifugation. Pellets were resuspended in lysis buffer for downstream DNA and RNA isolation. A full description of the FANS procedure is given in S1 Supporting Information.

### Isolation of DNA and total RNA from sorted nuclei

DNA was extracted from sorted nuclei with MasterPure Complete DNA and RNA Purification Kit (Epicentre), DNA purity was assessed on NanoDrop, and DNA concentration measured on Qubit (DNA HS assay). Further details are available in S1 Supporting Information. Lysates were thawed on ice and total RNA extracted with mirVana miRNA Isolation Kit (Ambion). Up-concentration was performed using RNA Clean & Concentrator-5 kit (Zymo Research). RNA concentration and integrity were assessed on Bioanalyzer with the RNA Pico Kit (Agilent Technologies). Further details are found in S1 Supporting Information.

### RRBS

A modified version of the gel-free protocol by Boyle *et al*. [43] was used for RRBS library preparation. The main changes to the protocol were inclusion of a two-sided size selection prior to bisulfite conversion, and sample pooling performed after completion of single libraries. A full description of the RRBS library prep and sequencing is given in S1 Supporting Information. Libraries were subjected to either 75 bp single read sequencing on NextSeq500 (Illumina), or 150 bp single read sequencing on HiSeq2500 (Illumina). For sequencing on NextSeq500, a pool of 14 libraries were run twice, with 50% PhiX spike-in at each run. On HiSeq2500, pools of 15 libraries were sequenced over two lanes, using 10% PhiX spike-in.

### High throughput mRNAseq

SMART-Seqv4 Ultra Low InputRNA Kit for Sequencing (Takara Bio) was used to amplify mRNA from total RNA, and the resulting cDNA was used as input in library preparation with ThruPlex DNAseq Kit (Rubicon Genomics). See S1 Supporting Information for details regarding cDNA synthesis and library preparation. Libraries were sequenced on NextSeq500 (75 bp single read), or HiSeq3000 (150 bp paired end). On NextSeq500, 12 libraries were pooled for one sequencing-run. The remaining 27 libraries were sequenced in one pool over three lanes on HiSeq3000.

### Computational methods

#### RRBS- and mRNAseq-post processing

Post-processing included trimming of reads using Trim Galore! v0.4.3 with parameters "—rrbs—illumina" and quality control with FastQC. Alignment to the reference mouse genome mm10 was performed with Bismark v0.20, powered by bowtie2. Quality metrics were collected from the resulting BAM files using the Picard tool CollectRrbsMetrics v2.18.15.

Alignment of the mRNAseq reads was accomplished with the Subread package through its R interface Rsubread, after trimming with Trim Galore! v0.4.3. Quality control of the resulting BAM files was undertaken with CollectRnaSeqMetrics from Picard v2.18.15. Uniquely mapped reads were assigned to genes and counted by the featureCounts function of Rsubread, using default parameters. As reference for the gene assignment we used release M16 of the comprehensive gene annotation of mm10 available from GENCODE. Only RNA aligning to mRNA regions was used for further analysis.

#### Annotation

The mouse genome build mm10 was used as reference in all analyses. Only autosomal data were analyzed. Coordinates of genes, exons and introns were taken from GENCODE's comprehensive annotation (www.gencodegenes.org/mouse/release_M16.html). The R package annotatr [44] was used to bioinformatically link CpG sites to the gene annotations.

Using predefined genomic features within annotatr [44], the promoter region for any gene was defined as the segment from -1kb upstream to the transcription start site and the upstream region from -5 kb to -1 kb, where negative numbers indicate positions upstream of transcription start site.

### Statistical methods

#### Analysis of DGE

The R package edgeR [45] was used to identify differentially expressed genes in the mRNAseq (mRNA) data set. The data from neuronal and glia cells were treated separately, contrasting KA versus SH samples in each case. Genes without official HGNC symbol were excluded from the analysis. Genes with low read counts were also removed, using the edgeR function filterByExpr with default parameters. After normalization to adjust for different library sizes (calcNormFactors) we followed a standard edgeR workflow to fit a quasi-likelihood negative binomial generalized log-linear model to the count data, and to perform the subsequent statistical analysis. A false discovery rate (FDR) approach was adopted to account for multiple testing, with a significance threshold of FDR 25%.

#### Analysis of DM

To identify loci exhibiting differential methylation between KA and SH samples, we adopted the edgeR workflow for RRBS data recently published by the edgeR authors [46]. Briefly, this approach entails treating the methylated and unmethylated counts at each locus as independent variables following a negative binomial distribution. As in the DGE analysis, DM analysis was carried out separately for neurons and glia cells, with an FDR of 25% as threshold for statistical significance. Before the analysis, filters were applied to all CpG sites where more than 10% of the samples had either very low coverage (< 8 reads) or excessively high coverage (> 99.5 quantile across all sites and samples). The DM analysis was performed both at the level of individual CpG sites, and in aggregated form within pre-defined genomic features: upstream, promoter, UTR5, exons, introns, gene body (i.e. the union of all exons and introns of a specific gene) and UTR3. For the aggregated analysis the input was the mean counts across all covered CpG's within the region.

#### Combined DM and DGE analysis

For each genomic feature (upstream, promoter, UTR5, exon, intron, gene body, UTR3), a combined analysis of DGE and DM was performed in order to unveil genes for which both methylation and gene expression differed significantly between the two groups. To reduce the statistical noise, the DGE analysis was reanalyzed for each genomic feature type, using only the relevant subset of the data. Specifically, for each genomic feature type, only the genes present in the aggregated DM data set were kept in the DGE analysis. Co-incidence of DGE and DM was declared for features surviving an FDR cutoff of 25% in both analyses.

#### Functional enrichment analysis

Enrichment analyses of Gene Ontology (GO) and Kyoto Encyclopedia of Genes and Genomes (KEGG) pathways were performed with the goana and kegga functions of edgeR, with the parameter species = "Mm". These functions conduct overlap tests for the up- and down-regulated DE genes, and for the genes overlapping DMRs.

### Quality control

#### Bisulfite conversion rate estimation

The conversion rate estimate computed by Picard/CollectRrbsMetrics is based on the conversion of non-CpG cytosines. As methylation of non-CpG cytosines is non-negligible in neuronal cells, this may bias the results. To account for this, we also performed an alternative estimate of the conversion rates directly from the untrimmed fastq files, by checking the methylation status specifically on the (unmethylated) cytosines added in the end-repair step of the RRBS preparation (private bash script). See S1 Supporting Information for further information of bisulfite conversion rate.

#### Multidimensional scaling

In order to validate our cell sorting procedures, and look for outliers among the samples, multidimensional scaling (MDS) plots were produced for the mRNAseq and RRBS data sets. The MDS computations were done by the plotMDS function of edgeR, selecting the top 100 most variable loci. The actual plots were created with ggplot2 [47].

#### Expression of neuronal and glial genes in NeuN+ and NeuN- fraction

Normalized counts for expression of neuronal (*RBFOX3*), glial (*ALDHL1L1*, *CX3CR1*, *MBP)*, *pericytal (PDGFRB) and endothelial (PECAM1*) genes were used to visualize enrichment of neurons in the NeuN+ fraction and glia in the NeuN- fraction.

### Selection of relevant GO and KEGG terms

Relevant (Tables 1 and 2) and epilepsy-relevant (Fig 2) GO and KEGG terms in neurons and glia were selected manually based on reviews on the subject [9] and personal knowledge. The list of GO and KEGG terms derived from our DGE analysis (for a full list see S1 Table) was manually filtered for specific terms and relevant up-/downregulated genes within these terms in neurons and glia selected for presentation.

**Table 1:** Differentially expressed genes in neurons at 24 hours post injection in the intracortical kainic acid model of mTLE.

Differentially expressed genes in neurons			
**Upregulated genes (N = 135)**			
**Gene symbol**	**logFC**	**FDR**	**Gene description**
Acan	3,94	0,000	aggrecan
Sdc1	3,60	0,001	syndecan 1
Inhba	4,12	0,001	inhibin beta-A
Socs3	4,21	0,001	suppressor of cytokine signaling 3
Timp1	5,38	0,007	tissue inhibitor of metalloproteinase 1
Megf11	2,25	0,011	multiple EGF-like-domains 11
Nptx2	3,61	0,011	neuronal pentraxin 2
Hspa1a	5,46	0,011	heat shock protein 1A
Fgl2	3,07	0,011	fibrinogen-like protein 2
Col27a1	3,22	0,012	collagen, type XXVII, alpha 1
Mapk4	2,28	0,012	mitogen-activated protein kinase 4
Cd1d1	2,94	0,018	CD1d1 antigen
Sik1	2,31	0,019	salt inducible kinase 1
Hspa1b	4,82	0,019	heat shock protein 1B
Tnc	2,08	0,019	tenascin C
Ptgs2	3,11	0,019	prostaglandin-endoperoxide synthase 2
Gipr	3,72	0,019	gastric inhibitory polypeptide receptor
Trib1	2,46	0,019	tribbles pseudokinase 1
Tpbg	2,10	0,019	trophoblast glycoprotein
Lhfp	1,71	0,019	lipoma HMGIC fusion partner
Fosb	3,32	0,019	FBJ osteosarcoma oncogene B
Arc	2,40	0,020	activity regulated cytoskeletal-associated protein
Fosl2	2,26	0,020	fos-like antigen 2
Gadd45g	2,54	0,027	growth arrest and DNA-damage-inducible 45 gamma
Pcdh11x	2,26	0,027	protocadherin 11 X-linked
Pmepa1	2,30	0,027	prostate transmembrane protein, androgen induced 1
Stk40	2,03	0,027	serine/threonine kinase 40
Pde6b	2,91	0,027	phosphodiesterase 6B, cGMP, rod receptor, beta polypeptide
Wisp1	2,09	0,027	WNT1-inducible-signaling pathway protein 1
9330188P03Rik	3,35	0,027	RIKEN cDNA 9330188P03 gene
Pappa	2,89	0,027	pregnancy-associated plasma protein A
Hspb1	4,00	0,027	heat shock protein 1
Atf3	4,52	0,029	activating transcription factor 3
Tll1	3,71	0,029	tolloid-like
Sulf1	1,57	0,031	sulfatase 1
Lbh	3,01	0,034	limb-bud and heart
Nedd9	1,51	0,035	neural precursor cell expressed, developmentally down-regulated gene 9
Parp3	2,93	0,035	poly (ADP-ribose) polymerase family, member 3
Rrad	4,89	0,035	Ras-related associated with diabetes
Trh	6,22	0,035	thyrotropin releasing hormone
4931440P22Rik	1,70	0,037	RIKEN cDNA 4931440P22 gene
Cyr61	2,90	0,037	Cysteine-rich angiogenic inducer 61
Fos	2,89	0,037	FBJ osteosarcoma oncogene
Cgref1	2,08	0,037	cell growth regulator with EF hand domain 1
Angptl4	2,49	0,037	angiopoietin-like 4
Srxn1	2,09	0,037	sulfiredoxin 1 homolog (S. cerevisiae)
Vim	2,84	0,039	vimentin
Vgf	2,37	0,041	VGF nerve growth factor inducible
Plpp4	2,33	0,043	phospholipid phosphatase 4
Clcf1	3,01	0,045	cardiotrophin-like cytokine factor 1
Zbtb46	1,58	0,048	zinc finger and BTB domain containing 46
Egr2	2,18	0,052	early growth response 2
Bach1	1,72	0,052	BTB and CNC homology 1, basic leucine zipper transcription factor 1
Samd4	1,90	0,052	sterile alpha motif domain containing 4
Rgs4	2,24	0,053	regulator of G-protein signaling 4
Cdkn1a	2,86	0,053	cyclin-dependent kinase inhibitor 1A (P21)
Adra1a	1,92	0,054	adrenergic receptor, alpha 1a
Csrnp1	2,36	0,054	cysteine-serine-rich nuclear protein 1
Gal	3,64	0,054	galanin and GMAP prepropeptide
Npas4	3,24	0,055	neuronal PAS domain protein 4
Sbno2	2,21	0,055	strawberry notch 2
Fndc9	3,19	0,061	fibronectin type III domain containing 9
Syndig1l	1,94	0,063	synapse differentiation inducing 1 like
Gpr3	1,97	0,075	G-protein coupled receptor 3
Fam129b	1,40	0,075	family with sequence similarity 129, member B
Sv2c	2,56	0,075	synaptic vesicle glycoprotein 2c
Adam19	1,62	0,081	a disintegrin and metallopeptidase domain 19 (meltrin beta)
Pim1	2,43	0,083	proviral integration site 1
Bag3	1,81	0,083	BCL2-associated athanogene 3
Sphk1	2,57	0,086	sphingosine kinase 1
Mapkapk3	1,97	0,086	mitogen-activated protein kinase-activated protein kinase 3
Zfp36	2,50	0,086	zinc finger protein 36
Cdh4	1,45	0,087	cadherin 4
Kdm6b	1,57	0,090	KDM1 lysine (K)-specific demethylase 6B
Emp1	2,49	0,091	epithelial membrane protein 1
Spp1	3,14	0,091	secreted phosphoprotein 1
Sorcs3	2,28	0,094	sortilin-related VPS10 domain containing receptor 3
Cd1d2	3,29	0,097	CD1d2 antigen
Prex1	2,06	0,101	phosphatidylinositol-3,4,5-trisphosphate-dependent Rac exchange factor 1
Pros1	2,10	0,101	protein S (alpha)
Uck2	1,35	0,101	uridine-cytidine kinase 2
Plce1	1,40	0,101	phospholipase C, epsilon 1
Tgfb1i1	1,66	0,101	transforming growth factor beta 1 induced transcript 1
Crispld2	2,18	0,117	cysteine-rich secretory protein LCCL domain containing 2
Frrs1	1,87	0,117	ferric-chelate reductase 1
Blnk	2,81	0,118	B cell linker
1700071M16Rik	1,68	0,119	RIKEN cDNA 1700071M16 gene
Rgs20	1,74	0,119	regulator of G-protein signaling 20
Ier2	2,17	0,120	immediate early response 2
Itprip	1,88	0,130	inositol 1,4,5-triphosphate receptor interacting protein
Smad7	1,83	0,130	SMAD family member 7
Svil	1,52	0,130	supervillin
Serinc2	1,79	0,136	serine incorporator 2
Cemip2	1,44	0,154	cell migration inducing hyaluronidase 2
Mir132	3,39	0,154	microRNA 132
Pear1	3,01	0,164	platelet endothelial aggregation receptor 1
Zdhhc22	1,85	0,167	zinc finger, DHHC-type containing 22
Medag	2,23	0,167	mesenteric estrogen dependent adipogenesis
Amotl1	1,71	0,175	angiomotin-like 1
Serpina3i	2,75	0,178	serine (or cysteine) peptidase inhibitor, clade A, member 3I
Ptgs1	2,01	0,178	prostaglandin-endoperoxide synthase 1
Ifit1	2,33	0,178	interferon-induced protein with tetratricopeptide repeats 1
Kcnip3	1,67	0,178	Kv channel interacting protein 3, calsenilin
Odc1	1,57	0,178	ornithine decarboxylase, structural 1
Igsf9b	2,27	0,178	immunoglobulin superfamily, member 9B
Homer1	1,49	0,179	homer scaffolding protein 1
Spred1	1,62	0,184	sprouty protein with EVH-1 domain 1, related sequence
Samd11	2,19	0,186	sterile alpha motif domain containing 11
Cdk18	1,98	0,186	cyclin-dependent kinase 18
Scd4	2,01	0,191	stearoyl-coenzyme A desaturase 4
Dgat2l6	3,15	0,191	diacylglycerol O-acyltransferase 2-like 6
Dusp4	1,88	0,191	dual specificity phosphatase 4
Anxa2	2,12	0,191	annexin A2
Serpina3n	1,85	0,191	serine (or cysteine) peptidase inhibitor, clade A, member 3N
Tspan9	1,68	0,191	tetraspanin 9
Eva1b	2,00	0,191	eva-1 homolog B (C. elegans)
Btc	2,40	0,191	betacellulin, epidermal growth factor family member
Acvr1c	1,91	0,193	activin A receptor, type IC
Rara	1,54	0,194	retinoic acid receptor, alpha
St8sia2	2,11	0,195	ST8 alpha-N-acetyl-neuraminide alpha-2,8-sialyltransferase 2
Tm4sf1	2,49	0,195	transmembrane 4 superfamily member 1
Cdh22	1,77	0,195	cadherin 22
Gfra1	1,50	0,202	glial cell line derived neurotrophic factor family receptor alpha 1
Itga5	2,11	0,205	integrin alpha 5 (fibronectin receptor alpha)
C2cd4b	2,11	0,206	C2 calcium-dependent domain containing 4B
Rasa4	1,84	0,220	RAS p21 protein activator 4
Mapk6	1,51	0,223	mitogen-activated protein kinase 6
Egr4	1,91	0,227	early growth response 4
Itpkc	1,84	0,227	inositol 1,4,5-trisphosphate 3-kinase C
Ptx3	2,72	0,235	pentraxin related gene
Tnfrsf12a	1,87	0,235	tumor necrosis factor receptor superfamily, member 12a
Drd1	1,82	0,246	dopamine receptor D1
**Downregulated genes (N = 15)**			
**Gene symbol**	**logFC**	**FDR**	**Gene description**
Cxcl12	-1,96	0,027	chemokine (C-X-C motif) ligand 12
Ogn	-2,71	0,029	osteoglycin
Plk5	-2,79	0,040	polo like kinase 5
Cys1	-1,85	0,041	cystin 1
Capn3	-2,14	0,052	calpain 3
Echdc2	-1,77	0,079	enoyl Coenzyme A hydratase domain containing 2
Cyp7b1	-2,28	0,097	cytochrome P450, family 7, subfamily b, polypeptide 1
Gm12216	-1,65	0,101	predicted gene 12216
Gstm6	-1,57	0,161	glutathione S-transferase, mu 6
Cd34	-1,63	0,167	CD34 antigen
Stxbp6	-1,51	0,186	syntaxin binding protein 6 (amisyn)
Crlf1	-1,97	0,194	cytokine receptor-like factor 1
Macrod1	-1,52	0,195	MACRO domain containing 1
Gm35339	-1,43	0,195	predicted gene, 35339
6330420H09Rik	-2,15	0,216	RIKEN cDNA 6330420H09 gene

Differentially expressed genes in neurons (FDR < 0.25); logFC = log fold change; FDR = false discovery rate.

**Table 2:** Differentially expressed genes in glia at 24 hours post injection in the intracortical kainic acid model of mTLE.

Differentially expressed genes in glia			
**Upregulated genes (N = 147)**			
**Gene symbol**	**logFC**	**FDR**	**Gene description**
Serpina3n	4,23	0,001	serine (or cysteine) peptidase inhibitor, clade A, member 3N
Thbd	2,93	0,001	thrombomodulin
Ch25h	5,14	0,001	cholesterol 25-hydroxylase
Lilr4b	4,88	0,002	leukocyte immunoglobulin-like receptor, subfamily B, member 4B
Gm3448	3,23	0,003	predicted gene 3448
Ucn2	8,54	0,003	urocortin 2
Ccl2	3,45	0,003	chemokine (C-C motif) ligand 2
Socs3	3,66	0,004	suppressor of cytokine signaling 3
Ier5l	2,55	0,005	immediate early response 5-like
Calca	4,68	0,005	calcitonin/calcitonin-related polypeptide, alpha
Sphk1	3,89	0,005	sphingosine kinase 1
Ecm1	2,40	0,005	extracellular matrix protein 1
Emp1	3,67	0,005	epithelial membrane protein 1
Ahnak2	3,87	0,005	AHNAK nucleoprotein 2
Spp1	4,71	0,005	secreted phosphoprotein 1
S1pr3	3,16	0,005	sphingosine-1-phosphate receptor 3
Fn1	2,64	0,007	fibronectin 1
Fgl2	2,97	0,008	fibrinogen-like protein 2
Timp1	4,60	0,008	tissue inhibitor of metalloproteinase 1
Tm4sf1	4,06	0,008	transmembrane 4 superfamily member 1
Rasgef1c	3,04	0,009	RasGEF domain family, member 1C
Ifit3	2,69	0,010	interferon-induced protein with tetratricopeptide repeats 3
Vgf	2,83	0,010	VGF nerve growth factor inducible
Il11	3,79	0,012	interleukin 11
Itga5	3,16	0,014	integrin alpha 5 (fibronectin receptor alpha)
Iigp1	3,09	0,015	interferon inducible GTPase 1
Hmga1b	2,26	0,015	high mobility group AT-hook 1B
Gadd45g	2,59	0,015	growth arrest and DNA-damage-inducible 45 gamma
Tnc	2,01	0,015	tenascin C
Cd44	3,16	0,015	CD44 antigen
Gpr151	3,26	0,017	G protein-coupled receptor 151
Ier3	2,44	0,020	immediate early response 3
Tnfrsf12a	2,69	0,020	tumor necrosis factor receptor superfamily, member 12a
Sv2c	3,05	0,021	synaptic vesicle glycoprotein 2c
Rasl11a	1,99	0,021	RAS-like, family 11, member A
Klk9	3,23	0,021	kallikrein related-peptidase 9
Btc	3,29	0,024	betacellulin, epidermal growth factor family member
Cebpd	1,95	0,025	CCAAT/enhancer binding protein (C/EBP), delta
Nptx2	2,91	0,025	neuronal pentraxin 2
Adam8	3,04	0,025	a disintegrin and metallopeptidase domain 8
Slc39a14	2,07	0,025	solute carrier family 39 (zinc transporter), member 14
Inhba	2,61	0,025	inhibin beta-A
Cdh22	2,38	0,025	cadherin 22
Fos	2,95	0,026	FBJ osteosarcoma oncogene
Rhoj	3,00	0,027	ras homolog family member J
Lilrb4a	4,02	0,027	leukocyte immunoglobulin-like receptor, subfamily B, member 4A
Cd300lf	3,60	0,028	CD300 molecule like family member F
Gadd45b	3,35	0,030	growth arrest and DNA-damage-inducible 45 beta
Cacng5	2,10	0,030	calcium channel, voltage-dependent, gamma subunit 5
Ifi204	4,09	0,032	interferon activated gene 204
Dab2	2,15	0,036	disabled 2, mitogen-responsive phosphoprotein
Myc	2,30	0,036	myelocytomatosis oncogene
Ifi207	3,11	0,043	interferon activated gene 207
Parp3	2,74	0,044	poly (ADP-ribose) polymerase family, member 3
Hspb1	3,54	0,046	heat shock protein 1
Trib1	2,04	0,047	tribbles pseudokinase 1
Rasip1	2,30	0,049	Ras interacting protein 1
Egr2	2,12	0,058	early growth response 2
Lpl	1,88	0,058	lipoprotein lipase
Tubb6	2,19	0,058	tubulin, beta 6 class V
Tpbg	1,72	0,058	trophoblast glycoprotein
Msr1	3,41	0,058	macrophage scavenger receptor 1
Sbno2	2,17	0,058	strawberry notch 2
Gcnt2	2,42	0,061	glucosaminyl (N-acetyl) transferase 2, I-branching enzyme
Fosb	2,69	0,061	FBJ osteosarcoma oncogene B
Serpine1	3,97	0,064	serine (or cysteine) peptidase inhibitor, clade E, member 1
Oasl2	2,47	0,064	2'-5' oligoadenylate synthetase-like 2
Srxn1	1,89	0,065	sulfiredoxin 1 homolog (S. cerevisiae)
Ptgs2	2,43	0,066	prostaglandin-endoperoxide synthase 2
Slc10a6	3,88	0,070	solute carrier family 10 (sodium/bile acid cotransporter family), member 6
Ahnak	1,95	0,072	AHNAK nucleoprotein (desmoyokin)
Nedd9	1,33	0,073	neural precursor cell expressed, developmentally down-regulated gene 9
Rai14	1,61	0,074	retinoic acid induced 14
Layn	1,95	0,075	layilin
Col16a1	2,51	0,076	collagen, type XVI, alpha 1
Atp10a	2,07	0,078	ATPase, class V, type 10A
Fstl4	1,87	0,078	follistatin-like 4
Wwtr1	1,58	0,080	WW domain containing transcription regulator 1
Gal	3,44	0,081	galanin and GMAP prepropeptide
Mx1	3,40	0,081	MX dynamin-like GTPase 1
Hmga1	2,18	0,091	high mobility group AT-hook 1
Irgm1	1,56	0,091	immunity-related GTPase family M member 1
Odc1	1,69	0,092	ornithine decarboxylase, structural 1
Gldn	3,04	0,093	gliomedin
Egr1	2,33	0,094	early growth response 1
Cchcr1	1,58	0,094	coiled-coil alpha-helical rod protein 1
Junb	2,29	0,102	jun B proto-oncogene
Slc5a3	1,82	0,102	solute carrier family 5 (inositol transporters), member 3
Socs2	1,76	0,102	suppressor of cytokine signaling 2
Il4ra	1,81	0,102	interleukin 4 receptor, alpha
Irf7	2,39	0,104	interferon regulatory factor 7
Nlrc5	2,21	0,104	NLR family, CARD domain containing 5
Ptx3	2,99	0,105	pentraxin related gene
Fgf18	2,32	0,107	fibroblast growth factor 18
Ifit3b	2,41	0,111	interferon-induced protein with tetratricopeptide repeats 3B
Strip2	1,74	0,115	striatin interacting protein 2
Has2	3,19	0,116	hyaluronan synthase 2
Mir212	4,52	0,117	microRNA 212
Flnc	3,71	0,118	filamin C, gamma
Map3k6	2,39	0,124	mitogen-activated protein kinase kinase kinase 6
Timeless	1,39	0,124	timeless circadian clock 1
Itga7	1,38	0,132	integrin alpha 7
Bcl3	3,83	0,134	B cell leukemia/lymphoma 3
Snhg15	1,56	0,134	small nucleolar RNA host gene 15
Ccl12	2,59	0,142	chemokine (C-C motif) ligand 12
Mamstr	2,09	0,142	MEF2 activating motif and SAP domain containing transcriptional regulator
Clcf1	2,36	0,142	cardiotrophin-like cytokine factor 1
Bdnf	1,81	0,142	brain derived neurotrophic factor
Ier2	2,03	0,142	immediate early response 2
Rnf138rt1	5,32	0,149	ring finger protein 138, retrogene 1
Fosl2	1,59	0,152	fos-like antigen 2
Slfn10-ps	2,78	0,160	schlafen 10, pseudogene
Amotl1	1,65	0,164	angiomotin-like 1
Mir132	3,30	0,174	microRNA 132
Serpina3i	2,64	0,174	serine (or cysteine) peptidase inhibitor, clade A, member 3I
Hmox1	1,87	0,174	heme oxygenase 1
Lrtm2	1,62	0,175	leucine-rich repeats and transmembrane domains 2
Spred3	1,72	0,175	sprouty-related EVH1 domain containing 3
Vmn1r15	6,73	0,175	vomeronasal 1 receptor 15
Rtp4	1,91	0,177	receptor transporter protein 4
Rnf125	2,28	0,177	ring finger protein 125
Slfn2	2,93	0,182	schlafen 2
Mchr1	1,73	0,185	melanin-concentrating hormone receptor 1
Piezo2	1,68	0,185	piezo-type mechanosensitive ion channel component 2
Anxa2	2,01	0,185	annexin A2
Gpd1	1,68	0,190	glycerol-3-phosphate dehydrogenase 1 (soluble)
Cyr61	2,08	0,194	Cysteine-rich angiogenic inducer 61
Plaur	2,39	0,194	plasminogen activator, urokinase receptor
Kdm6b	1,32	0,201	KDM1 lysine (K)-specific demethylase 6B
Ifit1	2,11	0,201	interferon-induced protein with tetratricopeptide repeats 1
Itga2b	1,93	0,202	integrin alpha 2b
Fgfr4	2,25	0,202	fibroblast growth factor receptor 4
Bst2	2,06	0,202	bone marrow stromal cell antigen 2
Gm6225	2,35	0,207	predicted gene 6225
Cbln4	1,60	0,208	cerebellin 4 precursor protein
Serpina3m	2,79	0,216	serine (or cysteine) peptidase inhibitor, clade A, member 3M
Akap12	1,34	0,218	A kinase (PRKA) anchor protein (gravin) 12
Sdc1	1,59	0,219	syndecan 1
Ndst1	1,59	0,219	N-deacetylase/N-sulfotransferase (heparan glucosaminyl) 1
Npas4	2,45	0,221	neuronal PAS domain protein 4
Tspan4	1,89	0,226	tetraspanin 4
Klk6	2,76	0,226	kallikrein related-peptidase 6
Cxcl10	2,90	0,226	chemokine (C-X-C motif) ligand 10
Col7a1	1,75	0,227	collagen, type VII, alpha 1
Plce1	1,17	0,237	phospholipase C, epsilon 1
Peak1	1,41	0,238	pseudopodium-enriched atypical kinase 1
Itga1	1,36	0,245	integrin alpha 1
**Downregulated genes (N = 15)**			
**Gene symbol**	**logFC**	**FDR**	**Gene description**
Aifm3	-2,53	0,005	apoptosis-inducing factor, mitochondrion-associated 3
Sowaha	-2,14	0,005	sosondowah ankyrin repeat domain family member A
Gdpd2	-2,73	0,012	glycerophosphodiester phosphodiesterase domain containing 2
Btbd17	-2,36	0,015	BTB (POZ) domain containing 17
Slc2a5	-2,59	0,015	solute carrier family 2 (facilitated glucose transporter), member 5
Pcx	-2,11	0,016	pyruvate carboxylase
Hapln1	-2,63	0,025	hyaluronan and proteoglycan link protein 1
Shroom2	-2,28	0,026	shroom family member 2
Gpr12	-2,22	0,039	G-protein coupled receptor 12
Ccdc13	-1,80	0,046	coiled-coil domain containing 13
Fn3k	-2,03	0,049	fructosamine 3 kinase
P2ry12	-2,57	0,053	purinergic receptor P2Y, G-protein coupled 12
Cygb	-1,88	0,053	cytoglobin
Ankub1	-2,23	0,060	ankrin repeat and ubiquitin domain containing 1
Siglech	-2,16	0,061	sialic acid binding Ig-like lectin H
Itpka	-1,70	0,061	inositol 1,4,5-trisphosphate 3-kinase A
Traf4	-1,81	0,065	TNF receptor associated factor 4
Hpca	-1,84	0,074	hippocalcin
Ppp1r1b	-1,75	0,077	protein phosphatase 1, regulatory inhibitor subunit 1B
Nkain4	-2,55	0,077	Na+/K+ transporting ATPase interacting 4
Folh1	-2,24	0,077	folate hydrolase 1
Kctd4	-2,09	0,081	potassium channel tetramerisation domain containing 4
Gstm6	-1,67	0,083	glutathione S-transferase, mu 6
Shisa8	-2,21	0,091	shisa family member 8
2810468N07Rik	-2,22	0,092	RIKEN cDNA 2810468N07 gene
Abca9	-1,97	0,092	ATP-binding cassette, sub-family A (ABC1), member 9
Paqr7	-1,93	0,099	progestin and adipoQ receptor family member VII
Chn1	-1,71	0,099	chimerin 1
Ntsr2	-2,14	0,105	neurotensin receptor 2
Myh14	-1,76	0,105	myosin, heavy polypeptide 14
Nwd1	-1,82	0,108	NACHT and WD repeat domain containing 1
Fam234a	-1,77	0,109	family with sequence similarity 234, member A
Susd5	-1,88	0,116	sushi domain containing 5
Faah	-1,50	0,117	fatty acid amide hydrolase
Tppp3	-1,76	0,124	tubulin polymerization-promoting protein family member 3
Abca6	-1,46	0,124	ATP-binding cassette, sub-family A (ABC1), member 6
Gnai1	-1,90	0,134	guanine nucleotide binding protein (G protein), alpha inhibiting 1
Cfap100	-1,48	0,135	cilia and flagella associated protein 100
Grm3	-2,01	0,142	glutamate receptor, metabotropic 3
Phgdh	-1,66	0,149	3-phosphoglycerate dehydrogenase
Selplg	-2,14	0,152	selectin, platelet (p-selectin) ligand
Epn2	-1,61	0,171	epsin 2
2900052N01Rik	-2,06	0,174	RIKEN cDNA 2900052N01 gene
Rlbp1	-1,78	0,175	retinaldehyde binding protein 1
Pantr1	-1,72	0,175	POU domain, class 3, transcription factor 3 adjacent noncoding transcript 1
Nat8f4	-1,42	0,175	N-acetyltransferase 8 (GCN5-related) family member 4
Plk5	-2,14	0,175	polo like kinase 5
Nat8f1	-1,91	0,175	N-acetyltransferase 8 (GCN5-related) family member 1
1700066M21Rik	-1,65	0,175	RIKEN cDNA 1700066M21 gene
Adi1	-1,61	0,176	acireductone dioxygenase 1
Tmem191c	-1,45	0,177	transmembrane protein 191C
Gmnc	-2,55	0,177	geminin coiled-coil domain containing
Zfp763	-1,51	0,182	zinc finger protein 763
Slc25a18	-1,79	0,185	solute carrier family 25 (mitochondrial carrier), member 18
Hhip	-2,01	0,185	Hedgehog-interacting protein
Calb1	-1,51	0,185	calbindin 1
Chst5	-1,74	0,185	carbohydrate (N-acetylglucosamine 6-O) sulfotransferase 5
Trim59	-2,18	0,185	tripartite motif-containing 59
Gpr34	-2,22	0,185	G protein-coupled receptor 34
Olfml1	-2,24	0,185	olfactomedin-like 1
Mturn	-1,41	0,185	maturin, neural progenitor differentiation regulator homolog (Xenopus)
Gstm1	-1,80	0,185	glutathione S-transferase, mu 1
Enho	-1,63	0,185	energy homeostasis associated
Prodh	-1,86	0,185	proline dehydrogenase
Slc27a1	-1,71	0,185	solute carrier family 27 (fatty acid transporter), member 1
Pacsin3	-1,44	0,185	protein kinase C and casein kinase substrate in neurons 3
Htr1a	-1,95	0,185	5-hydroxytryptamine (serotonin) receptor 1A
Dll3	-1,72	0,187	delta like canonical Notch ligand 3
Map6d1	-1,60	0,187	MAP6 domain containing 1
Prrg1	-1,61	0,193	proline rich Gla (G-carboxyglutamic acid) 1
Carns1	-1,88	0,201	carnosine synthase 1
Tle2	-1,48	0,201	transducin-like enhancer of split 2
Macrod1	-1,45	0,202	MACRO domain containing 1
Nrgn	-1,51	0,204	neurogranin
Plin3	-2,18	0,207	perilipin 3
Grhpr	-1,38	0,208	glyoxylate reductase/hydroxypyruvate reductase
Sult1a1	-2,19	0,214	sulfotransferase family 1A, phenol-preferring, member 1
Pls1	-1,58	0,216	plastin 1 (I-isoform)
Lin7b	-1,69	0,216	lin-7 homolog B (C. elegans)
Armh4	-1,53	0,218	armadillo-like helical domain containing 4
Panx2	-1,33	0,226	pannexin 2
Appl2	-1,76	0,227	adaptor protein, phosphotyrosine interaction, PH domain and leucine zipper containing 2
Grhl1	-1,01	0,227	grainyhead like transcription factor 1
Tmem255b	-1,65	0,233	transmembrane protein 255B
Pigz	-1,71	0,243	phosphatidylinositol glycan anchor biosynthesis, class Z

Differentially expressed genes in glia (FDR < 0.25); logFC = log fold change; FDR = false discovery rate.

## Results

### Quality control

Bisulfite conversion rates were above 98% (S1 Supplementary Information and S3 Fig) and multidimensional scaling plots of mRNAseq and RRBS data sets distinguished clearly between neurons and glia (S4 Fig and S5 Fig). The NeuN+ fraction enriched for neuronal and the NeuN- fraction for glial mRNA (S6 Fig), indicating a successful separation of neurons and glia.

### Differential methylation

A statistical analysis of Differentially Methylated CpGs (DM CpGs) compared right hippocampi of KA to SH mice at 24 hours post injection. After filtering, 928 430 CpG sites remained and were used in subsequent analyses. On average, across all CpG sites and all samples, each CpG was covered by 29.8 reads. In individual samples the mean read depth varied from 20.0 to 35.2 (median = 30.6, interquartile range = [27.4–32.7]).

#### Differentially methylated sites

**Fig 1:**
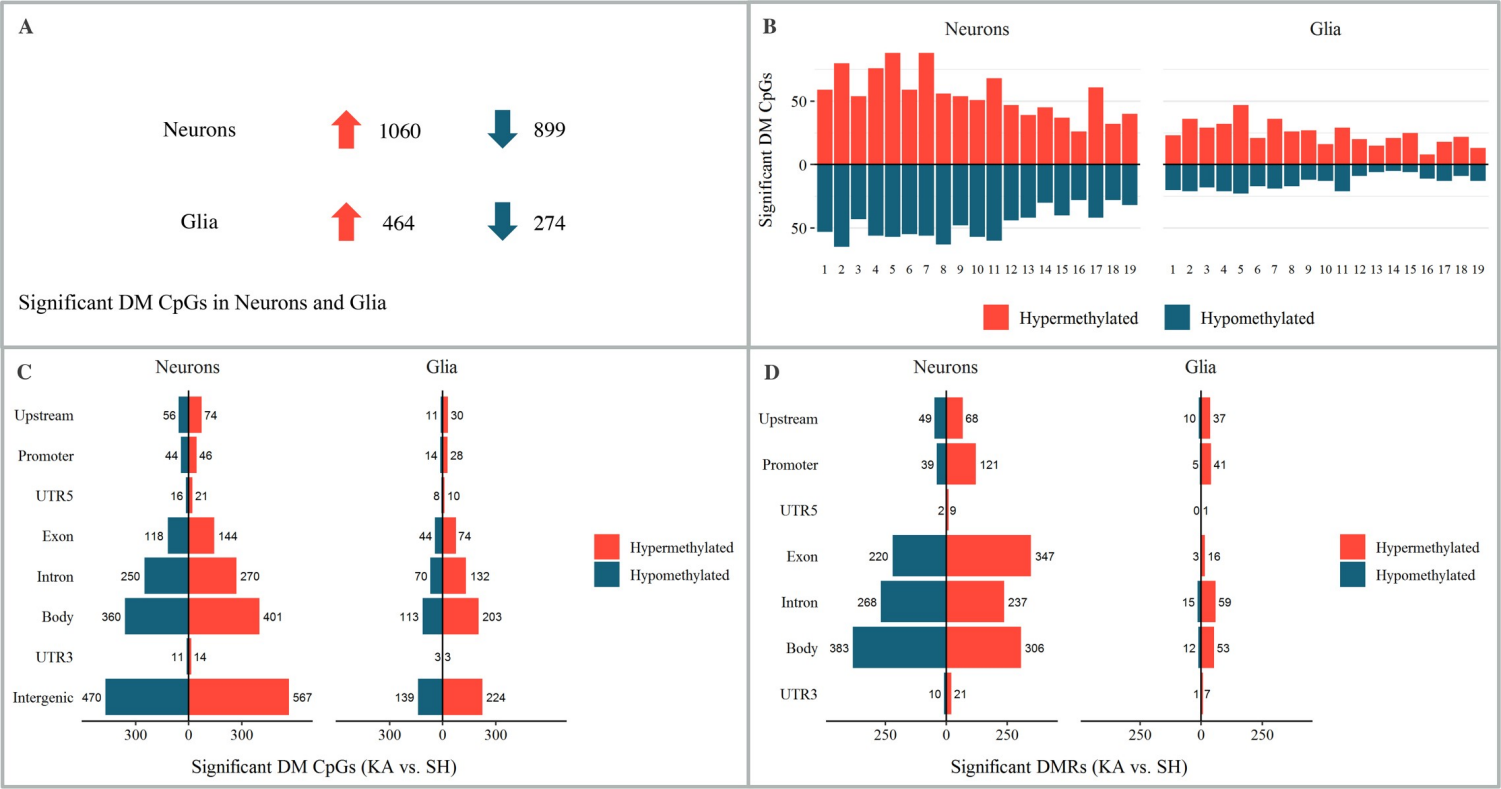
Differential DNA methylation at 24 hours post injection in the intracortical kainic acid model of mTLE. (A)–(C) Statistical analysis of differentially methylated CpGs (DM CpGs) of KA versus SH group, 24 hours post injection. (A) DM CpGs in neurons and glia; upward arrow indicates hypermethylation and downward arrow hypomethylation of DM CpGs. (B) Chromosomal distribution of DM CpGs. (C) Distribution of DM CpGs amongst genomic features. (D) Distribution of differentially methylated regions (DMR) amongst genomic features.

The analysis of significantly altered DM CpGs revealed 1060 hyper- and 899 hypomethylated (ratio 1.2:1) CpG sites in neurons and 464 hyper- and 274- hypomethylated (ratio: 1.7:1) CpG sites in glia (Fig 1). Most of the DM CpGs localized to either gene bodies or intergenic regions (for full list see supplementary 2) and were distributed evenly across chromosomes (sex chromosomes excluded), apart from a possible higher ratio of hyper-/hypomethylated CpGs at chromosome 13 to 15 in glia. The ratio of hypermethylated to hypomethylated CpG sites was highest at upstream (1.3:1) and intergenic (1.2:1) regions for neurons and upstream (2.7:1) and promoter (2.0:1) for glia. For detailed information including GO and KEGG annotation of DM CpGs see S1 Table.

#### Differentially methylated CpG sites common to both neurons and glia

Neurons and glia shared four commonly hypermethylated (fraction: 0.3%) and zero commonly hypomethylated DM CpGs. One DM CpG was hypermethylated in neurons and hypomethylated in glia (fraction: 0.0%) and one DM CpGs hypomethylated in neurons and hypermethylated in glia (fraction: 0.0%). Three of the four commonly hypermethylated DM CpGs localized to gene bodies and one to an upstream region of the associated gene. The other two CpGs were associated with intergenic regions.

#### Differentially methylated regions

In order to obtain information about genomic features with significantly altered differential methylation, an aggregated analysis was conducted for each genomic feature (upstream, promoter, UTR5, exon, intron, gene body, UTR3) separately. Most DMR were found in gene bodies (e.g. exonic and intronic areas) and promoters in both neurons and glia. The greatest hyper-/hypomethylated regions ratio was found at 5’UTRs (ratio 4.5:1), gene bodies (ratio 3.7:1) and promoters (ratio 3.1:1) in neurons and at promoters (ratio 8.2:1), 3’UTRs (ratio 7.0:1) and exons (ratio 5.3:1) for glia. For a full list of significantly altered DMR and their annotated GO and KEGG terms see S1 Table.

### Differential gene expression

**Fig 2:**
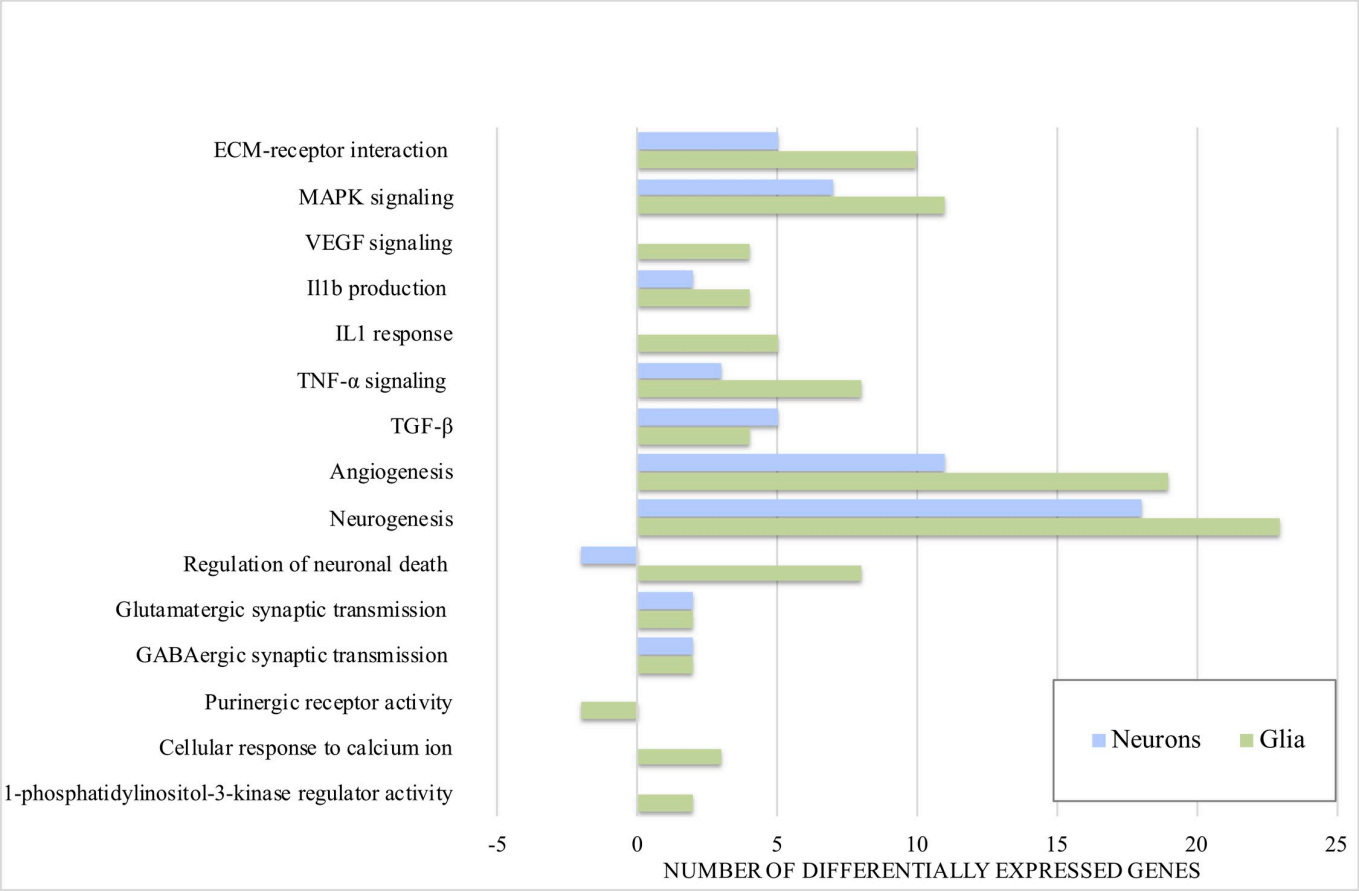
Number of up- and downregulated neuronal and glial genes within epilepsy-relevant functional annotation terms (GO / KEGG). Neuronal and glial contribution to epilepsy-relevant GO / KEGG pathways (p<0.05) amongst differentially expressed genes. Negative values indicate downregulated genes, positive values upregulated genes.

DGE analysis compared right hippocampi of KA to SH at 24 hours post injection. After filtering, 23369 genes were used for downstream analysis. After processing, alignment and filtering, the mRNASeq samples yielded on average 7.5 giga bases (Gb) data aligned to the mouse genome (median = 9.0 Gb; range = [2.4 Gb—13.0 Gb]). The fraction aligning specifically to mRNA regions varied from 16% to 41.2% (median = 28.5), resulting in an average of 2.2 Gb per sample informative for DGE analysis (median = 2.2 Gb; range = [0.4 Gb—4.3 Gb]). In neurons, 135 genes were up- and 15 downregulated (Table 1), while in glia 147 genes were up- and 85 downregulated (Table 2). A relevant selection of broader GO / KEGG terms is presented in Tables 3 (neurons) and 4 (glia). For neuronal and glial contribution to epileptogenesis in terms of number of differentially expressed genes as part of epilepsy-relevant GO / KEGG terms, see Fig 2. For a detailed list of up- and downregulated genes and associated GO / KEGG terms see S1 Table.

**Table 3:** Relevant GO and KEGG terms of up- and downregulated genes in neurons at 24 hours post injection in the intracortical kainic acid model of mTLE.

	Upregulated genes in neurons (N = 135)	Downregulated genes in neurons (N = 15)
GO*	• Cell differentiation	• Lymphocyte migration
	Signal transduction	• Leukocyte migration
	• Cell death	• Endothelial cell proliferation
	• Regulation of gene expression	• Regeneration
	• Cell-cell signaling	• Cellular response to DNA damage stimulus
	• Cell surface receptor signaling	• Growth factor activity
	• Cell growth	• Vesicle organization
KEGG*	• ECM-receptor interaction	
	• MAPK signaling	
	• IL17 signaling	
	• cAMP signaling	
	• TNF signaling	
	• VEGF signaling	
	• TGFbeta signaling	

Relevant GO and KEGG terms associated with significantly (FDR 0.25) upregulated genes

* = p<0.05.

**Table 4:** Relevant GO and KEGG terms of up- and downregulated genes in glia at 24 hours post injection in the intracortical kainic acid model of mTLE.

	Upregulated genes in glia (N = 147)	Downregulated genes in glia (N = 85)
GO*	• Regulation of IL1	• Purinergic nucleotide receptor activity
	• Response to IL1	• Regulation of ion transport
	• MAPK	• PLC activating G-protein receptor
	• Apoptotic process	• NAD binding
	• Lymphocyte chemotaxis	• Glutamate receptor signaling
	• Cytokine production	• Myelin sheath
	• Angiogenesis	• Mitochondrial part
KEGG*	• ECM-receptor interaction	• Glutathione metabolism
	• MAPK signaling	• Pyruvate metabolism
	• PI3K-Akt signaling pathway	• ABC transporters
	• Cytokine-cytokine receptor interaction	• cAMP signaling pathway
	• TNF signaling	• Glycine, serine and threonine metabolism
	• VEGF signaling	
	• JAK-stat signaling	

Relevant GO and KEGG terms associated with significantly (FDR 0.25) upregulated genes

* = p<0.05.

#### Genes up- or downregulated in both neurons and glia

A total number of 45 genes were upregulated in both neurons and glia. GO terms of these included “positive regulation of transcription” and “cytokine mediated signaling” whilst KEGG terms contained “ECM receptor interaction”, “VEGF-signaling” and “TNF-signaling”. Three genes were downregulated in both neurons and glia.

### Association between differential methylation and differential gene expression

An association between DM and DGE was calculated by alignment of significantly altered DMR and differentially expressed genes (Combined FDR 0.25, for details see S1 Table and S7–S20 Figs). No general correlation between DMR and DGE at various genomic features (upstream, promoter, UTR5, exon, intron, UTR3) was found (S1 Table and S7–S20 Figs). However, significant DMR coincided with DGE at 41 loci for neurons and 12 loci for glia (Tables 5 and 6).

**Table 5:** Association between DMR and DGE in neurons at 24 hours post injection.

	Neurons			
Genomic feature	Gene symbol	DM logFC	DGE logFC	Gene description
Upstream	*ZBTB18*	3,22	-1,20	Zinc finger and BTB domain-containing protein 18
	*SPP1*	-0,99	3,14	Osteopontin
Promoter	*SPP1*	-0,97	3,14	Osteopontin
	*PDE6B*	-2,72	2,91	Rod cGMP-specific 3',5'-cyclic phosphodiesterase subunit beta
	*BTG2*	5,23	1,49	Protein BTG2
	*DPYSL3*	4,70	1,02	Dihydropyrimidinase-related protein 3
	*GADD45G*	4,59	2,54	Growth arrest and DNA damage-inducible protein GADD45 gamma
	*SCRT2*	3,77	1,43	Transcriptional repressor scratch 2
Exon	*TRIB2*	4,72	1,20	Tribbles homolog 2
	*ZFP36*	1,77	2,50	mRNA decay activator protein ZFP36
	*GAL*	2,13	3,64	Galanin
	*CXCL12*	-0,87	-1,96	Stromal cell-derived factor 1
	*SV2C*	-1,27	2,56	Synaptic vesicle glycoprotein 2C
	*GM5577*	3,67	-1,36	Predicted gene 5577
	*LTBP1*	-1,11	1,48	Latent transforming growth factor beta binding protein 1
	*ARL4D*	-0,61	1,61	ADP-ribosylation factor-like 4D
	*FNDC9*	-1,07	3,19	Fibronectin type III domain-containing protein 9
	*CD34*	3,18	-1,63	Hematopoietic progenitor cell antigen CD34
	*EPHX1*	1,99	-1,28	Epoxide hydrolase 1
	*TUBB6*	-0,51	1,49	Tubulin beta-6 chain
	*BACH1*	-1,04	1,72	Fanconi anemia group J protein homolog
Intron	*SAMD11*	0,73	2,19	sterile alpha motif domain containing 11
	*KIF18A*	0,91	1,46	Kinesin-like protein KIF18A
	*NPTX2*	-0,69	3,61	Neuronal pentraxin-2
	*IGF2BP2*	-0,56	2,04	Insulin-like growth factor 2 mRNA-binding protein 2
	*NPTX2*	-0,69	3,61	Neuronal pentraxin-2
Gene body	*KIF18A*	0,80	1,46	Kinesin family member 18A
	*GAL*	2,09	3,64	Galanin peptides
	*TRIB2*	2,07	1,20	tribbles pseudokinase 2
	*SAMD11*	0,70	2,19	Sterile alpha motif domain-containing protein 11
	*ADGRF4*	0,92	1,66	Adhesion G protein-coupled receptor F4
	*LTBP1*	-0,52	1,48	Latent-transforming growth factor beta-binding protein 1
	*FAM129B*	-0,59	1,40	Niban-like protein 1
	*IGF2BP2*	-0,54	2,04	insulin-like growth factor 2 mRNA binding protein 2
	*CD34*	3,30	-1,63	CD34 antigen
	*SORCS3*	-0,40	2,28	VPS10 domain-containing receptor SorCS3
	*GXYLT2*	-0,85	1,40	Glucoside xylosyltransferase 2
	*SV2C*	-0,63	2,56	Synaptic vesicle glycoprotein 2C
	*ARL4D*	-0,60	1,61	ADP-ribosylation factor-like protein 4D
UTR3	*EGR3*	3,45	1,61	Early growth response protein 3
	*NEDD9*	-1,46	1,51	Enhancer of filamentation 1

Genetic loci with coincidence of significant DM and DGE (FDR 0.25); logFC = log fold change.

**Table 6:** Association between DMR and DGE in glia at 24 hours post injection.

	Glia			
Genomic feature	Gene symbol	DM logFC	DGE logFC	Gene description
Upstream	*ZBTB46*	-1,01	0,87	Zinc finger and BTB domain-containing protein 46
	*KIRREL2*	3,75	-1,41	Kin of IRRE-like protein 2
	*PANTR1*	-4,55	-1,72	POU3F3 Adjacent Non-Coding Transcript 1 (non coding RNA)
	*ASCL1*	-4,05	-1,42	Achaete-scute homolog 1
Promoter	*HDAC11*	4,28	-1,42	Histone deacetylase 11
	*DRD1*	4,24	1,62	D(1A) dopamine receptor
Exon	*ZFP467*	0,47	-1,38	Zinc finger protein 467
Intron	*DRD1*	4,17	1,62	D(1A) dopamine receptor
	*2810468N07RIK*	-3,95	-2,22	RIKEN cDNA (lncRNA)
Gene body	*-*			
UTR3	*FGFBP3*	3,75	-1,07	Fibroblast growth factor-binding protein 3

Genetic loci with coincidence of significant DM and DGE (FDR 0.25); logFC = log fold change.

## Discussion

Neurons and glial cells execute specific complementary tasks in normal brain functioning as well as in the pathological processes precipitating neurological diseases [9, 48, 49]. This diversity is represented on the transcriptome- [36, 50] and epigenome level [34, 35]. In this study we investigated neuronal and glial alterations of DNAm and gene expression and their possible association in a mouse model of epilepsy. In order to explore the epigenetic and transcriptomic signature of both cell types in early epileptogenesis, we separated neurons and glia by FANS [51]. This approach was recently applied in epigenetic studies [39–41]. We identified specific neuronal and glial DNAm and DGE changes at particular genomic loci, potentially including important upstream mechanisms worth further investigation.

### Differential methylation

With an overlap of only 0.22% of DM CpGs between neurons and glia, differential methylation occurs primarily in a cell specific manner during early epileptogenesis. Accordingly, the attributed GO terms to DM CpGs and DMR reveal mostly neuronal and glia specific terms (S1 Table). Apart from a near even ratio at neuronal promoters, we observe an overweight of hypermethylated to hypomethylated CpG sites at 24 hours post injection. In early stages of epileptogenesis, previous studies suggest no general alteration of DNAm [30] or a tendency towards hypomethylation [52]. In chronic phases of epilepsy, both hyper- and hypomethylation have been reported [29, 53]. Differences in pro-convulsant agents, mode and region at which they are applicated, stage of epileptogenesis, anatomical regions investigated, as well as the number of CpG sites covered, may account for varying results. A comparison of genes associated with significant DM CpGs and DMR from our study with results from hippocampal tissue from chronic TLE patients [54] unvails marginal but interesting overlaps. This may be due to different methylation analysis methods and stage specific character of hippocampal DNAm during epileptogenesis. A comparison of our results to those from peripheral blood of TLE patients [55], reveals 15 overlapping DM CpGs and 11 overlapping DMRs.

### Differential gene expression

Most differentially expressed genes detected in this study, were specific to either neurons or glia (S1 Table). Only 45 genes were commonly up- and three downregulated in neurons and glia. At this early stage of epileptogenesis, glial cells apparently contribute a higher number of altered gene transcripts than neurons within epilepsy-related GO and KEGG terms (Fig 2). Many significantly differentially expressed genes found in this epilepsy model overlap with data from other experimental and human TLE studies [29, 56–58]. Comparing our differentially expressed genes in neurons and glia with differential gene expression from various epilepsy models and stages of TLE [58], glia contributes with more upregulated gene transcripts (13 glia and 11 in neurons), supporting the notion that glia contributes essentially to epileptogenesis [59–66]. Neurons contribute with slightly higher number of altered gene transcripts when comparing to a amygdala stimulation model of epilepsy while glia contributes with a higher number of differentially expressed genes when comparing to a traumatic brain injury model of epilepsy [29]. Within up- and downregulated gene transcripts from a study on refractory human TLE, neurons and glia exhibit an almost even number of gene transcripts [57]. For an overview of essential up- and downregulated pathways see Tables 1 and 2. A summary of neuronal and glial contribution (number of genes within a GO / KEGG term) to epileptogenesis is presented in Fig 2.

### Altered epilepsy-relevant pathways based on differentially expressed genes

*Upregulation of growth arrest and DNA-damage-inducible beta/gamma* (GADD45B/G). One of the genes upregulated in both neurons and glia is *GADD45G*, a member of the environmental stress inducible *GADD45*-like genes that mediate activation of various pathways, including c-Jun N-terminal protein kinase family of mitogen-activated protein kinases [67]. It has previously been shown to be elevated after KA induced status epilepticus [68] and to possess DNA demethylation qualities [69], thus potentially linking epileptic activity to changes in DNA methylation. We also find elevated mRNA levels of *GADD45B*, which is another member of the *GADD45*-like genes. GADD45B has recently been shown to promote neuronal activity induced neurogenesis via demethylation of the BDNF and fibroblast growth factor promoter, linking neuronal activity to DNA methylation alterations [69].

### *Upregulation of sphingosine-kinase 1* (SPHK1) *and sphingosine 1 receptor 3* (S1R3)

Another interesting finding is the upregulation of *SPHK1* mRNA in neurons and glia. SPHK1 phosphorylates sphingosine to sphingosine-1-phosphate (S1P) [70]. S1P in turn is involved in neural development, signaling, autophagy and neuroinflammation as well as a plethora of pathological central nervous conditions [71] and has been shown to modulate histone deacetylase activity [72]. A recent study revealed antiepileptogenic effects of fingolimod [71], a SP1-receptor modulator and FDA approved drug for the treatment of multiple sclerosis [73], possibly via attenuation of astro- [74] and microglial [75] reactions. Further, we find elevated expression of *S1R3*, a S1P receptor, in glia. This receptor has been shown to be elevated in hippocampi of kainic acid and pilocarpine epilepsy models as well as in humans with TLE, and is mainly expressed in astrocytes [76].

### Upregulated mitogen-activated protein kinase (MAPK) pathways in neurons and glia

MAPK is a type of protein kinase specific to the amino acids serine and threonine. MAPKs are involved in directing cellular responses to a variety of different stimuli, such as proinflammatory cytokines, mitogens, osmotic stress and heat shock. They regulate cell functions including proliferation, gene expression and differentiation, mitosis, cell survival and apoptosis [77]. We find several genes within MAPK pathways (GO / KEGG) to be expressed more in the KA than SH group with glia contributing a higher number of differentially expressed genes within the pathways than neurons (S1 Table). In the context of epilepsy, MAPK are thought to play a role in Cx43 phosphorylation, involving TNF-α, interleukin (IL)-1b [78] and VEGF [79]. We find elevated expression levels of genes within KEGG pathways for both TNF-α (mostly in glia) and VEGF (slightly more in neurons, see S1 Table). Phosphorylation of Cx43 in turn has been associated with its elevated internalization and degradation [80], possible contributing to astrocyte uncoupling in both mTLE mice and humans [15].

*Astrocytic calcium signaling pathways altered in glia*. Neuronal activity induced elevations in astrocytic intracellular calcium levels may in turn facilitate astrocytic release of neuroactive substances including glutamate, aggravating epileptic activity [81]. In acute stages of epileptogenesis, calcium transients in astrocytes are increased, possibly contributing to elevated extracellular potassium levels via Calcium-dependent protease induced cleavage of the dystrophin associated protein complex [82, 83]. Elevated extracellular potassium levels in turn may lead to increased excitability of neurons and thereby generate epileptiform activity [84]. We also find elevated gene expression of inositol 1,4,5-trisphosphate 3-kinase A (*ITPKA*), a protein kinase inactivating inositol triphosphate dependent calcium release from the astrocyte endoplasmic reticulum [85, 86], in glia. Further, we find 1-Phosphatidylinositol-4,5-bisphosphate phosphodiesterase epsilon-1 (*PLCE1*), a member of the phosphatidylinositol-specific phospholipase C family that via G-protein coupled receptors are involved in Inositol-triphosphate and diacylglycerol generation and as such mediate intracellular Calcium elevation [87], elevated in glia. Thirdly, we find *CACNG5*, a calcium permissive AMPA receptor subunit [88], elevated in glia. Possibly all three genes mediate pro-epileptogenic effects via astrocytic calcium signaling. For a complete summary of differentially expressed genes see S1 Table.

### Relationship between differential methylation and differential gene expression

We did not find a general correlation of DM and DGE at specific genomic regions (S1 Table, S7–S20 Figs). However, DM coincided with DGE at 41 genes for neurons and 10 genes for glia. Our results are in line with previous studies that did not find a general correlation between DNA methylation and gene expression in epilepsy, but rather a number of singular genes where significant DM with DGE coincided [30, 89, 90]. Other studies did report a certain degree of general association between DM at specific genomic regions and DGE [29]. For a full list of coinciding alterations in DM and DGE see Table 5. The identified coinciding alterations of DM and DGE in this study are relatively few and their role in epileptogenesis remains uncertain. Interestingly, they point to genes and pathways previously implicated in epilepsy, TLE and epileptogenesis. In the following we present a selection of these in depth.

#### Coinciding alterations of differential methylation and differential gene expression in neurons

*Osteopontin* (SPP1) *promoter hypomethylation associated with elevated gene expression*. SPP1 mediates diverse aspects of cellular functioning in the central nervous system, e.g. the recruitment and activation of microglia and astrocytes, the cumulative effect possibly being neuroprotective [91]. In multiple sclerosis, SPP1 mediates pro-inflammatory pathways contributing to the relapse remission phenotype via e.g. NF-κB [92]. In our study, *SPP1* mRNA is significantly upregulated in both neurons and glia, and in neurons this elevated gene expression is associated with significant hypomethylation at the associated upstream region and promoter. These results confirm previous findings of elevated *SPP1* mRNA levels in epilepsy [93]. We further found elevated mRNA levels of *CD44*, a Osteopontin receptor [94] involved in epileptogenesis [95], in glia.

#### *Hypermethylation at a Galanin* (GAL) *exon associated with elevated gene expression*

*GAL* encodes the neuropeptide Galanin which previously has been shown to possess seizure attenuating properties and discussed as a possible antiepileptogenic target [96]. Further, in a recent study, a de novo mutation in GAL has been unveiled as a possible cause for TLE [97].

In our study, *GAL* mRNA is significantly upregulated in both glia and neurons (slightly higher log fold change (logFC) and lower FDR in neurons) and we find a significant association of hypermethylation of an exonic region of *GAL* with elevated gene expression in neurons. Thus, the hypermethylation at the exonic region of *GAL* with possible consecutive elevated levels of GAL might represent a crucial endogenic seizure attenuating mechanism.

### *Hypomethylation at synaptic vesicle protein 2 c* (SV2C) *exon associated with upregulated gene expression*

SV2C is, together with SV2A and SV2B, part of the family of synaptic vesicle proteins that are involved in Ca^2+^ dependent synaptic vesicle exocytosis and neurotransmission [98]. The most prominent epilepsy-related member, SV2A, is the main target through which levetiracetam and brivaracetam exert their antiepileptic and possibly antiepileptogenic effects [99]. In hippocampi of patients with chronic TLE, SV2C was the only of three synaptic vesicle proteins found to be significantly elevated. It was associated with mossy fiber sprouting and glutamatergic synapses and was proposed as a potential antiepileptogenic target [100]. Recent findings suggest a role of SV2C in the disruption of dopamine signaling in Parkinson’s Disease [101]. At 24 hours post injection, we find elevated levels of *SV2C* mRNA in glia and neurons. In neurons this elevated gene expression is associated with significant hypomethylation of its exonic regions. Hypomethylation of the *SV2C* exon may thus exert upstream pro-epileptogenic effects.

### Coinciding alterations of differential methylation and differential gene expression in glia

*Promoter hypermethylation at* HDAC11 *associated with reduced gene expression levels*. In line with previous results [102], we find *HDAC11* mRNA levels decreased after SE. This reduced gene expression coincides with a significantly increased methylation at its associated promoter in glia. Reduced levels of *HDAC11* may cause an increased acetylation at H4 [103], previously shown to correlate with elevated levels of c-fos, c-jun and BDNF [104]. BDNF in turn has been associated with seizure-aggravating effects in acute phases of epileptogenesis [105] and higher levels of the microRNA miR-132, possibly via ERK and MAPK pathways [106]. Further, miR-132 has recently been associated with seizure induced neuronal apoptosis [107]. We find elevated expression levels of *BDNF* (only glia), miR-132 (both neurons and glia) and MAPK- (glia more transcripts than neurons) and ERK- (glia more than neurons) pathways in early epileptogenesis. The hypermethylation of the *HDAC11* promoter with its possible downstream effects ultimately leading to elevated levels of *BDNF* and miR-132 might represent a possible antiepileptogenic target for site specific alteration of DNAm.

#### *Hypermethylation at the intron and promoter at dopamine receptor D1 (DRD1) associated with elevated* DRD1 *mRNA levels*

Dopamine exerts its seizure inducing effects via DRD1 mediated ERK1/2 pathways [108]. We find elevated levels of *DRD1* mRNA in both neurons and glia. In glia, this augmentation in gene expression is associated with significant hypermethylation in the intronic region and hypermethylation at the promoter of *DRD1*. Hypermethylation at glial *DRD1* (intronic region) may facilitate epileptogenesis.

### Technical limitations

This study features several technical limitations worth mentioning. Firstly, we cannot rule out that the NeuN- fraction contains a minor number of non-glial cells (pericytes, endothelial cells) [109, 110]. RRBS associated technical limitations involve loss of information associated with e.g. msp1 enzyme cleavage, library pooling/ fragment size selection, bisulfite conversion, sequencing (depth/coverage) [43]. The use of nuclear mRNA results in enrichment of mRNA coding for proteins with nuclear functions [111] and a potentially lower level of immediate early genes [112]compared to when using cytosolic mRNA [113].

## Conclusion

In this study, we found DNAm and DGE in early epileptogenesis to occur primarily in a cell-specific manner. We identified several potential neuronal and glial upstream targets worth further investigation. Information on the cellular origin of epigenomic and transcriptomic effects increases our understanding of involved pathological processes and provides a basis for possible future cell specific therapeutic approaches.

## Supporting information

S1 Table DM and DGE in neurons and glia. Table of significantly altered mRNA (RRBS) and DNA methylation (DM CpGs and DMR) in neurons and glia as well as overviews and adjunct functional annotations (GO / KEGG).
(XLSX)

S1 Supporting Information Detailed methods. (DOCX)

S1 Fig Flowchart of tissue processing from hippocampi to NeuN+ / NeuN- nuclei. Hippocampi from Kainate (n = 8) or Sham (n = 8) animals at 24 hrs. after injection were pooled (sample 1: pooled 4 to 1; sample 2 and 3: pooled 2 to 1) and homogenized to obtain single nuclei. The nuclei were filtered, centrifugated, pelleted and resuspended, before being subjected to FANS.
(TIF)

S2 Fig **Sorting of NeuN-positive and NeuN-negative nuclei by flow cytometry (A–F) Nuclei were defined as PI-positive events, and aggregated nuclei were excluded in an SSC-w vs FSC-a plot.** Single nuclei from a tissue not expressing NeuN (adult mouse liver) were used to define the NeuN-positive and NeuN-negative gates (A), and hippocampal nuclei were sorted accordingly (B).
(TIF)

S3 Fig Estimated bisulfite conversion rates. The left panel shows the PCT_NON_CPG_BASES_CONVERTED metric computed by Picard/CollectRRBSMetrics. This is defined as the fraction of converted cytosines among all non-CpG cytosines encountered in the sequencing data. The right panel shows the observed conversion rate of the unmethylated "end-repair" cytosines added in the RRBS prep (see methods for details).
(TIF)

S4 Fig Principal component analysis (MDS) of RRBS-Data. The principal component analysis of RRBS data distinguishes clearly between neurons and glia but not between KA and SH.
(TIF)

S5 Fig Principal component analysis (MDS) of RNA-Data. Principal component analysis of mRNAseq data clearly distinguished between neurons and glia as well as KA and SH.
(TIF)

S6 Fig Expression levels (mRNAseq, normalized counts) for CNS cell type specific genes. Neurons: *RBFOX3* (= NeuN), Astrocytes: *ALDH1L1*, Microglia: *CX3CR1*, Oligodendrocytes: *MBP*, Pericytes: *PDGFRB*, Endothelial cells: *PECAM1*; Expression in the NeuN+ and NeuN- fraction on the left and right side of each graph.
(TIF)

S7 Fig DM and DGE (neurons, upstream). Visualization of DM and DGE (FDR 0.25) for neurons (upstream). Genes associated with significantly altered DMR and DGE are indicated in the figure.
(TIF)

S8 Fig DM and DGE (glia, upstream). Visualization of DM and DGE (FDR 0.25) for glia (upstream). Genes associated with significantly altered DMR and DGE are indicated in the figure.
(TIF)

S9 Fig DM and DGE (neurons, promoter). Visualization of DM and DGE (FDR 0.25) for neurons (promoters). Genes associated with significantly altered DMR and DGE are indicated in the figure.
(TIF)

S10 Fig DM and DGE (glia, promoter). Visualization of DM and DGE (FDR 0.25) for glia (promoters). Genes associated with significantly altered DMR and DGE are indicated in the figure.
(TIF)

S11 Fig DM and DGE (neurons, UTR5). Visualization of DM and DGE (FDR 0.25) for neurons (UTR5). Genes associated with significantly altered DMR and DGE are indicated in the figure.
(TIF)

S12 Fig DM and DGE (glia, UTR5). Visualization of DM and DGE (FDR 0.25) for glia (UTR5). Genes associated with significantly altered DMR and DGE are indicated in the figure.
(TIF)

S13 Fig DM and DGE (neurons, exon). Visualization of DM and DGE (FDR 0.25) for neurons (exon). Genes associated with significantly altered DMR and DGE are indicated in the figure.
(TIF)

S14 Fig DM and DGE (glia, exon). Visualization of DM and DGE (FDR 0.25) for glia (exon). Genes associated with significantly altered DMR and DGE are indicated in the figure.
(TIF)

S15 Fig DM and DGE (neurons, intron). Visualization of DM and DGE (FDR 0.25) for neurons (intron). Genes associated with significantly altered DMR and DGE are indicated in the figure.
(TIF)

S16 Fig DM and DGE (glia, intron). Visualization of DM and DGE (FDR 0.25) for glia (intron). Genes associated with significantly altered DMR and DGE are indicated in the figure.
(TIF)

S17 Fig DM and DGE (neurons, gene body). Visualization of DM and DGE (FDR 0.25) for neurons (gene body). Genes associated with significantly altered DMR and DGE are indicated in the figure.
(TIF)

S18 Fig DM and DGE (glia, gene body). Visualization of DM and DGE (FDR 0.25) for glia (gene body). Genes associated with significantly altered DMR and DGE are indicated in the figure.
(TIF)

S19 Fig DM and DGE (neurons, UTR3). Visualization of DM and DGE (FDR 0.25) for neurons (UTR3). Genes associated with significantly altered DMR and DGE are indicated in the figure.
(TIF)

S20 Fig DM and DGE (glia, UTR3). Visualization of DM and DGE (FDR 0.25) for glia (UTR3). Genes associated with significantly altered DMR and DGE are indicated in the figure.
(TIF)
