# Supplementary material for: Neuronal and glial DNA methylation and gene expression changes in early epileptogenesis
Source: PLoS One. 2019 Dec 30;14(12):e0226575. doi: 10.1371/journal.pone.0226575 (PMC6936816; doi:10.1371/journal.pone.0226575)
Supplement: S1 Supporting Information — (DOCX) [file pone.0226575.s002.docx]

**FANS sorting of nuclei**
All solutions and tubes were pre-chilled on ice prior to use, samples kept on ice whenever possible, and centrifugations performed at 4 °C. Mixing or resuspension of samples/nuclei was done by gentle pipetting with a 1 mL tip.

**Reagents and solutions**

The following reagents were used: Sucrose (Sigma-Aldrich cat.no. S0389), Tricine (Sigma-Aldrich cat. no. T0377), KCl (VWR cat. no. 26764.298), MgCl_2_•6H_2_O (Merck Millipore cat. no. 1058330250), CaCl_2_•2H_2_O _(_Sigma-Aldrich cat. no. C7902), Protease Inhibitor Cocktail (Biotools cat. no. B14001), Triton X-100 (Sigma Aldrich cat. no. T8532), Debris Removal Solution (DRS) (Miltenyi cat.no. 130-109-398), DPBS (ThermoFisher Scientific cat.no. 14190144), anti-NeuN Alexa Fluor488 (anti-NeuN-488) (Millipore cat. No. MAB377X), propidium iodide 100 µg/mL (PI) (Miltenyi, cat.no. 130-093-233), mirVana lysis buffer (RNA lysis buffer) (from mirVana miRNA Isolation Kit, Ambion cat. no. AM 1560), Tissue and Cell Lysis Solution (DNA lysis buffer) (from MasterPure Complete DNA and RNA Purification Kit, Epicentre cat. no. MC85200).

Incubation Buffer (IB):

0.25M sucrose, 25 mM KCl, 5 mM MgCl_2_, 20 mM Tricine-KOH, pH 7.8

Homogenization buffer (HB):
IB supplemented with Triton X-100 to 0.1% and 1x Protease Inhibitor Cocktail

**Homogenization of tissue**
Tubes of pooled hippocampi hemispheres were put on ice for immediate processing. Per tube, 1 mL of HB was added, hippocampi allowed to thaw for 1 min, and tissue with buffer poured into a GentleMACS C Tube (Miltenyi cat.no. 130-093-237). C tubes were subject to the m_brain_02_01 program on a GentleMACS dissociator (Miltenyi) and spun down at 100 x g for 30 sec to pellet undissolved tissue, and the homogenate was filtered through a 70 μm filter into a 15 mL conical tube. 1 mL HB was added to the undissolved tissue, the m_brain_02_01 program repeated, the C Tube spun down, homogenate collected as above into the 15 mL tube, after which the process was repeated again. Finally, the filter was washed with 1 mL IB, leaving approximately 3.5 mL of nuclei suspension per sample. 20 µL of suspension was diluted with IB to 100 µL and used for calculating the total number of nuclei by counting on NucleoCounter NC-100 (Chemometec). The average number of nuclei per homogenized hippocampal hemisphere was 830.000. In parallel with the hippocampal samples a NeuN-negative control sample containing 50 mg of adult mouse liver was processed in order to facilitate gating in downstream nuclear sorting. This sample was run on GentleMACS using the m_liver_03 program.

**Debris removal**The exact volume of each sample was measured and 290 μL of DRS added per mL of nuclei suspension. After gentle mixing, samples were overlaid with 4 mL DPBS and spun at 3000 x g for 20 min, with slow acceleration and brake turned off. Upper layer, debris interphase, and lower layer were discarded, and nuclear pellets were resuspended in 100 μL IB per one million nuclei (based on counting on filtered homogenate, see Homogenization of tissue) and transferred to 5 mL polypropylene tubes.

**Staining of nuclei**

Anti-NeuN-488 was added to each tube to a final concentration of 0.1 µg/mL, and samples incubated for 1 h on ice in the dark. Samples were not washed prior to sorting, as additional centrifugation resulted in a significant loss of nuclei.

**Nuclear sorting**

Sorting of nuclei was performed on a BD FACSAria (BD Biosciences). 0.1 µg PI (DNA stain) was added to each sample approximately five minutes prior to sorting. The instrument was set to gently agitate the sample at 100 rpm. The following strategy was used for gating (S 2 Fig): 1) A nuclear gate was defined by PI-positive events (excitation by 488 nm laser, emission collected by 616/23 band-pass filter). 2) Aggregated nuclei were excluded in a dot plot using the pulse width of side scatter (SSC-w) versus the pulse area of forward scatter (FSC-a). 3) The NeuN-negative gate was set based on signal from anti-NeuN-488 stained liver sample (excitation by 488 nm laser, emission collected by 530/30 band-pass filter). NeuN-positive and NeuN-negative hippocampal nuclei were sorted into 5mL tubes prefilled with 2 mL DPBS. On average, 31% of nuclei present in the filtered homogenate were recovered after sorting, with 66% sorted as NeuN-positive, and 34% sorted as NeuN-negative. Mean purity of the sorted fractions was measured to 93% and 95% for the positive and negative population, respectively. Immediately after sorting 3 μL of 1 M MgCl_2_ and 5 μL of 1 M CaCl_2_ were added per mL of nuclei suspension (in order to avoid nuclear disruption during centrifugation, as suggested by (Jiang, 2008 #100), followed by gentle mixing. Half of each sample was transferred to a new tube, and all tubes were spun at 500 x g for 5 min. Supernatants were discarded, and nuclear pellets resuspended as follows (per sorted hippocampal sample): One pellet of NeuN-negative nuclei in 100 µL DNA lysis buffer, one pellet of NeuN-positive nuclei in 200 µL DNA lysis buffer, one pellet of NeuN-negative nuclei in 500 µL RNA lysis buffer, and one pellet of NeuN-positive nuclei in 500 µL RNA lysis buffer. Lysates for DNA isolation were stored at -20 °C until time of extraction. Lysates for RNA isolation were stored at -80 °C, with RNA extraction performed within 24 h.

**Isolation of total RNA from sorted nuclei**
Lysates were thawed on ice and total RNA extracted according to procedures E and F.I in the mirVana miRNA Isolation Kit protocol (Ambion cat. no. AM 1560). Up-concentration was performed using the RNA Clean & Concentrator-5 kit (Zymo Research cat.no. R1015), followed by elution in 8 µL nuclease free water. RNA concentration and integrity were assessed on Bioanalyzer (Agilent Technologies) with the RNA Pico Kit (Agilent Technologies cat.no. 5067-1513). Mean yield from NeuN-positive and NeuN-negative nuclei was approximately13 ng and 1ng per sorted hemisphere. Mean RNA Integrity number (RIN) was 7.9.

**DNA isolation from sorted nuclei**

DNA was extracted from sorted nuclei with MasterPure Complete DNA and RNA Purification Kit (Epicentre cat.no. MC85200). First, lysates were thawed in room temperature, mixed with Proteinase K (0.33 µL per 100 µL lysate), and DNA extracted following the DNA purification protocol for cell samples, starting at step 5. DNA was resuspended in 20 μL of 5 mM Tris-Cl. DNA purity was assessed on NanoDrop (ThermoFisher Scientific), showing mean 260/280 and 230/280 absorbance ratios of 1.88 and 1.15, respectively. DNA concentration was measured on Qubit (DNA HS assay, ThermoFisher Scientific cat.no. Q32851). Mean yield from NeuN-positive and NeuN-negative nuclei was 460 ng (positives) and 150 ng (negatives) per sorted hemisphere.

**RRBS**

A modified version of the gel-free protocol by [1] was used for RRBS library preparation. The main changes to the protocol were inclusion of a two-sided size selection prior to bisulfite conversion, and sample pooling performed after completion of single libraries. 100ng DNA was digested with 20 units of MspI (New England Biolabs cat.no. R0106S) in NEB buffer 2 (New England Biolabs cat.no M0212S) at 37°C for 24 hours, followed by gap filling and A-tailing using 5 units of Klenow fragment (NEB cat.no. M0212S) and 1 mM dCTP, 1 mM dGTP, and 10 mM dATP (NEB cat.no. N0446S), with incubation at 30°C for 20 min followed by 37°C for 20 min. Clean-up was performed using 3 volumes of AmPure XP beads (Beckman Coulter cat.no. A63880). For this step, and all downstream use of AmPure beads, care was taken not to over-dry the beads prior to elution in 10 mM Tris-Cl. 1 µL of a 1:10 dilution of adaptors from Illumina kit FC-121-3001/2 or FC-121-4001/2 was used for indexing of samples, with ligation performed by T4 ligase (NEB cat.no. M0202S) at 16°C for 19 h. A two-sided size selection using AmPure XP beads was performed aiming to recover ligation products between approximately 200 and 400 bp. The exact volume of ligation reaction was measured, and fragments longer than 400 bp was removed by to consecutive rounds of binding to beads. First, a sample:bead ratio of 1:0.4 was used (e.g. 30 µL ligation reaction and 12 µL beads). After 15 min incubation and 5 min on magnet, the supernatant was collected and the beads discarded. This step was repeated, adding 0.2 x initial sample volume to the supernatant (6 µL beads if ligation reaction was 30 µL), discarding the beads after incubation, and keeping the supernatant. To remove fragments shorter than 200 bp, a standard AmPure XP clean-up was performed, adding a bead volume corresponding to 1.25 x initial sample volume, minus the volume of bead solution already present in the supernatant (e.g. 30 µL x 1.25 – (6+12) µL, i.e. 19.5 µL bead volume added). Size selected fragments were bisulfite converted with the EpiTect Bisulfite Kit (Qiagen cat.no. 59104) using the procedure for FFPE samples and two consecutive rounds of the incubations given in the protocol. Bisulfite converted samples were eluted in 20 µL elution buffer. 16 µL of the eluate was mixed with 89.6 µL nuclease free water, 16 µL 10mM dNTPs (ThermoFisher scientific cat.no. N8080260), 16 µL 10× PfuTurbo Cx buffer, 9.6 µL of 2.5 µM TruSeq forward primer (5’AATGATACGGCGACCACCGAGAT 3’), 9.6 µL of 2.5 µM TruSeq reverse primer (5’CAAGCAGAAGACGGCATACGA 3’), and 3.2 µL PfuTurbo Cx hotstart DNA polymerase (Agilent cat.no. 600412). The mix was divided into eight aliquots in a 96 well PCR plate, and PCR was performed at the following conditions: 95°C for 2 min, followed by 16 cycles of 95°C 30 sec, 65°C 30 sec, 72°C 45 sec, and finally 72°C for 7 min. The aliquots were pooled together, and the PCR reaction purified by 1.25 x volume of AMPure XP beads, with elution in 22 µL 10 mM Tris-Cl. Fragment distribution was analyzed on Bioanalyzer, using the DNA HS kit (Agilent cat.no. 5067-4626). Libraries showing adapter dimers on the Bioanalyzer traces went through a second AMPure XP clean-up (sample:bead ratio 1:1.25) and run again on Bioanalyzer. Library concentration was measured on Qubit (DNA HS assay).

**High throughput mRNA-sequencing**

SMART-Seqv4 Ultra Low Input RNA Kit for Sequencing (Takara Bio cat.no. 634891) was used for mRNA specific cDNA synthesis and amplification through oligo(dT) priming, using an input of 1 ng total RNA and 10 cycles of PCR. Two samples had limited amounts of RNA, and a lower input amount was used (approximately 0.05 ng (15 cycles PCR)). cDNA concentrations were measured on Qubit (DNA HS assay), and 20 ng of each sample was fragmented on Covaris LE220 (Covaris) using the recommended settings in the SMART-Seqv4 protocol. Fragmented cDNA was purified with 2 volumes AMPure XP beads, eluted in 12 µL 10 mM Tris-Cl, and measured on Qubit.
Sequencing libraries were prepared from fragmented cDNA using the ThruPlex DNA-seq Kit (Rubicon Genomcs cat.no. R400407) according to manufacturer’s instructions. Library concentration was measured on Qubit, and fragment distribution analyzed on D1000 Screen Tape on TapeStation (Agilent cat.no. 5067-5582/3).

**Bisulfite conversion rates**

Bisulfite conversion rates. All RRBS samples showed estimated conversion rates above 98% (S3 Fig). Of note, the Picard/CollectRRBSMetrics tool consistently gave lower estimates for neuronal samples than for glial samples. This is an expected consequence of the well-known fact that neurons, unlike other cells (in mammals), contain non-negligible methylation of cytosines outside of CpG context [2]. Indeed, what CollectRRBSMetrics measures is the fraction of non-CpG cytosines which are converted; this will under-estimate the true conversion rates when used on neuron samples. Our alternative method for estimating the conversion rates, using unmethylated cytosines added in the library prep, did not show any systematic difference between neurons and glial cells.

**References**

1. Boyle P, Clement K, Gu H, Smith ZD, Ziller M, Fostel JL, et al. Gel-free multiplexed reduced representation bisulfite sequencing for large-scale DNA methylation profiling. Genome Biology. 2012;13(10):R92.

2. Kozlenkov A, Roussos P, Timashpolsky A, Barbu M, Rudchenko S, Bibikova M, et al. Differences in DNA methylation between human neuronal and glial cells are concentrated in enhancers and non-CpG sites. Nucleic Acids Research. 2014;42(1):109-27.
