# Supplementary figures and images for: Neuronal and glial DNA methylation and gene expression changes in early epileptogenesis
Source: PLoS One. 2019 Dec 30;14(12):e0226575. doi: 10.1371/journal.pone.0226575 (PMC6936816; doi:10.1371/journal.pone.0226575)

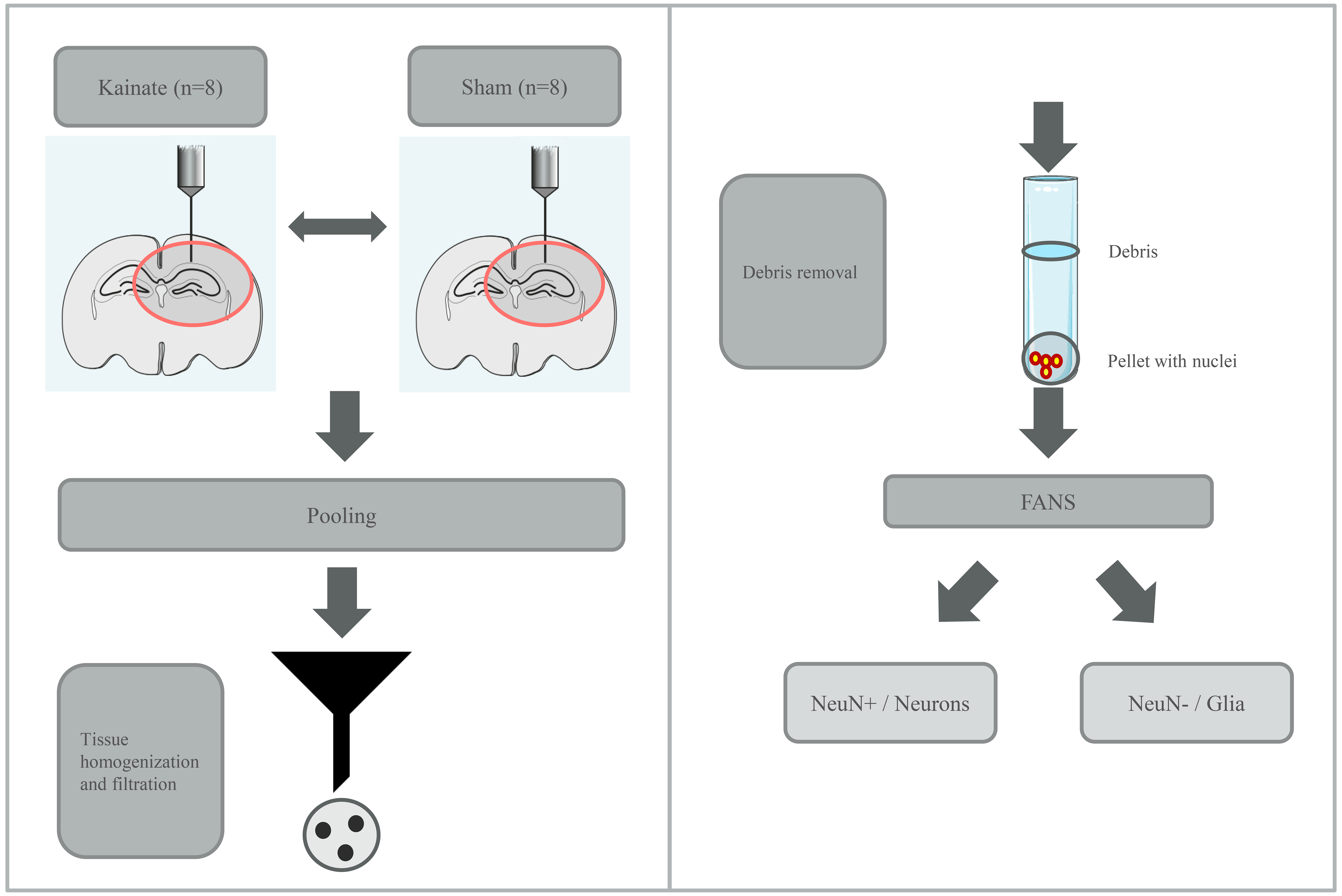

Supplement: S1 Fig — Hippocampi from Kainate (n = 8) or Sham (n = 8) animals at 24 hrs. after injection were pooled (sample 1: pooled 4 to 1; sample 2 and 3: pooled 2 to 1) and homogenized to obtain single nuclei. The nuclei were filtered, centrifugated, pelleted and resuspended, before being subjected to FANS. (TIF) [file pone.0226575.s003.tif]

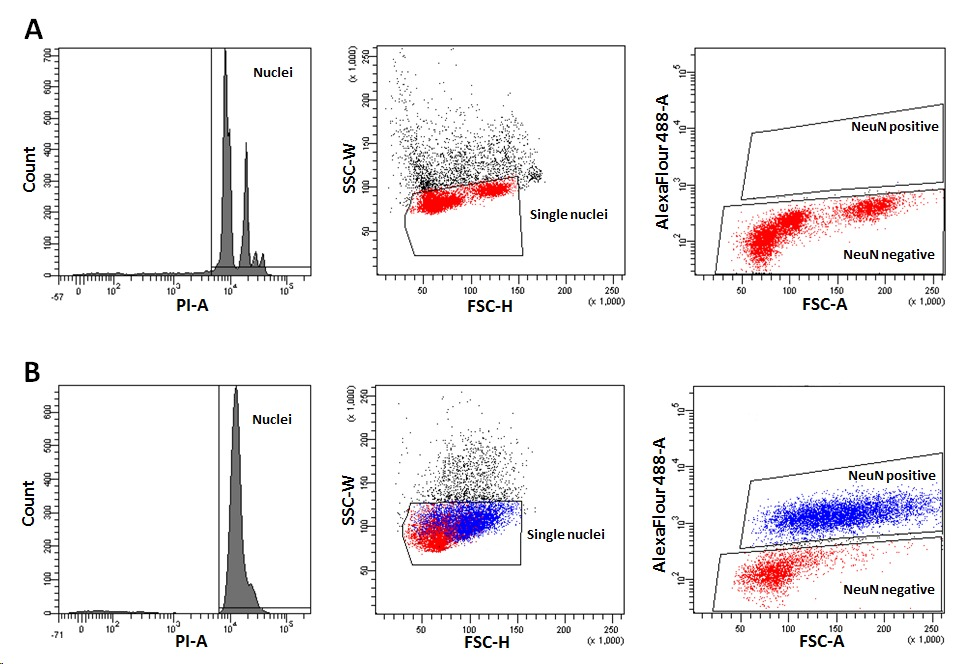

Supplement: S2 Fig — Sorting of NeuN-positive and NeuN-negative nuclei by flow cytometry (A–F) Nuclei were defined as PI-positive events, and aggregated nuclei were excluded in an SSC-w vs FSC-a plot. Single nuclei from a tissue not expressing NeuN (adult mouse liver) were used to define the NeuN-positive and NeuN-negative gates (A), and hippocampal nuclei were sorted accordingly (B). (TIF) [file pone.0226575.s004.tif]

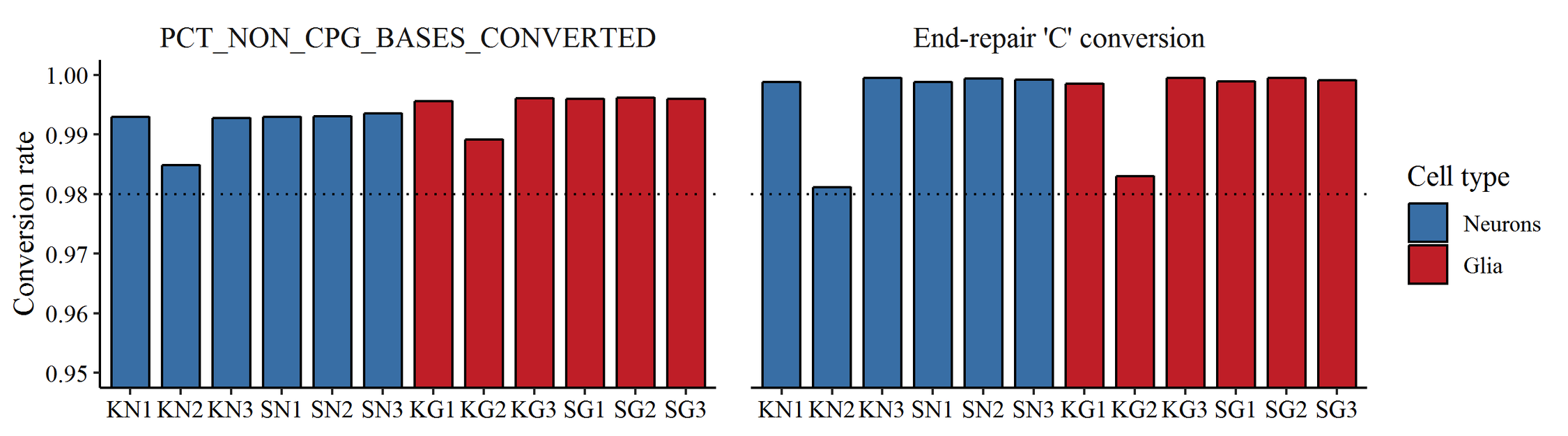

Supplement: S3 Fig — The left panel shows the PCT_NON_CPG_BASES_CONVERTED metric computed by Picard/CollectRRBSMetrics. This is defined as the fraction of converted cytosines among all non-CpG cytosines encountered in the sequencing data. The right panel shows the observed conversion rate of the unmethylated "end-repair" cytosines added in the RRBS prep (see methods for details). (TIF) [file pone.0226575.s005.tif]

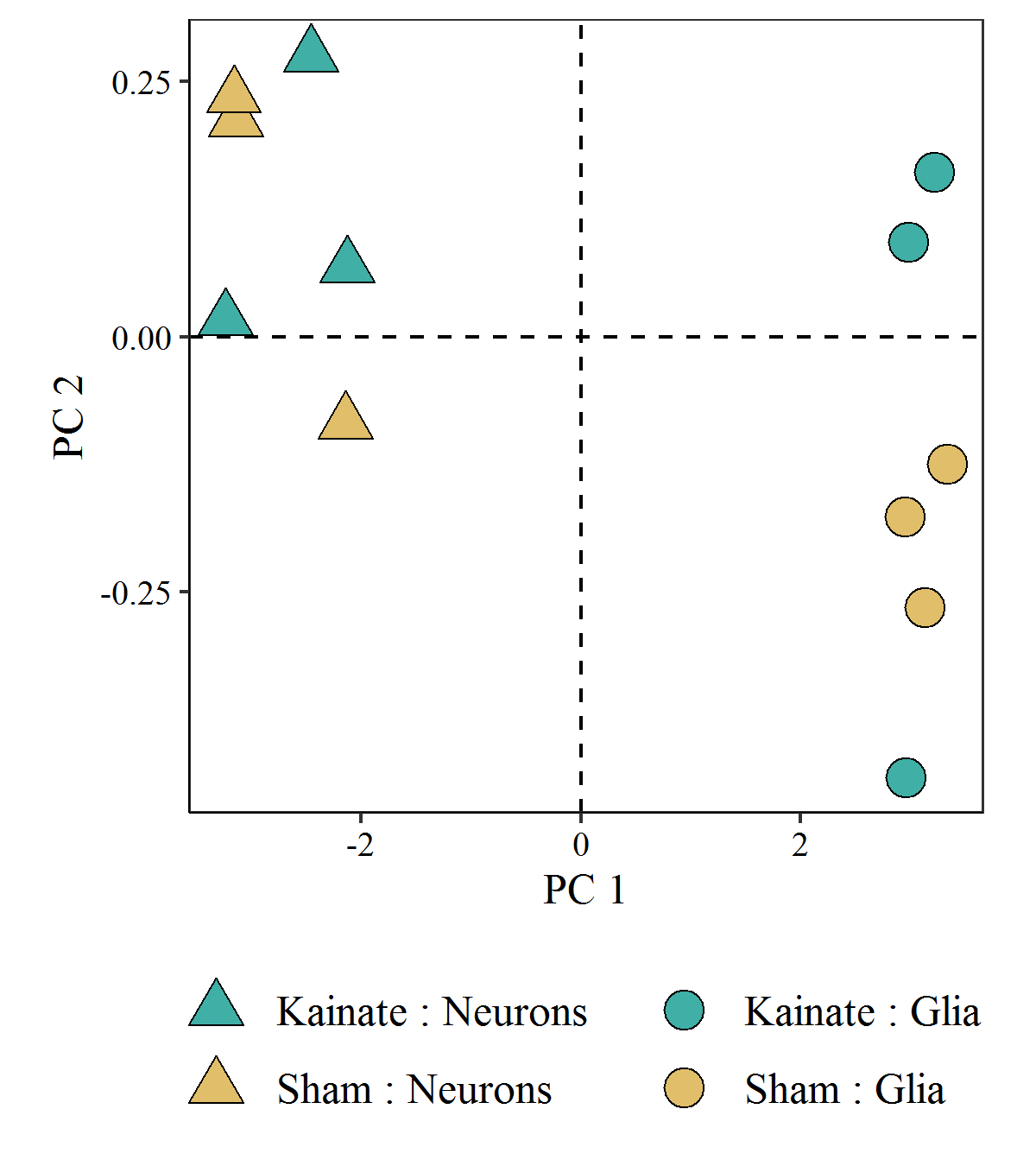

Supplement: S4 Fig — The principal component analysis of RRBS data distinguishes clearly between neurons and glia but not between KA and SH. (TIF) [file pone.0226575.s006.tif]

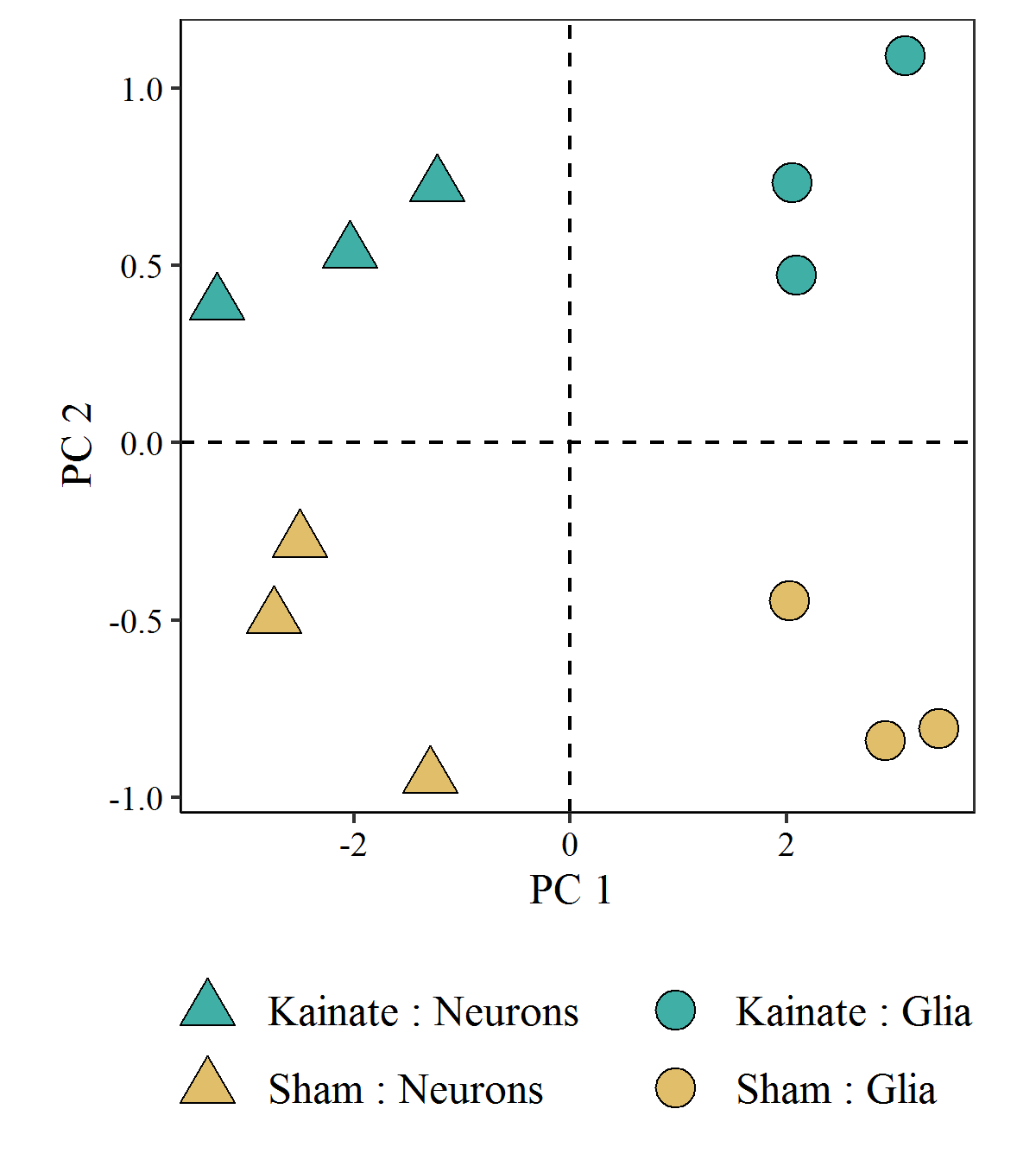

Supplement: S5 Fig — Principal component analysis of mRNAseq data clearly distinguished between neurons and glia as well as KA and SH. (TIF) [file pone.0226575.s007.tif]

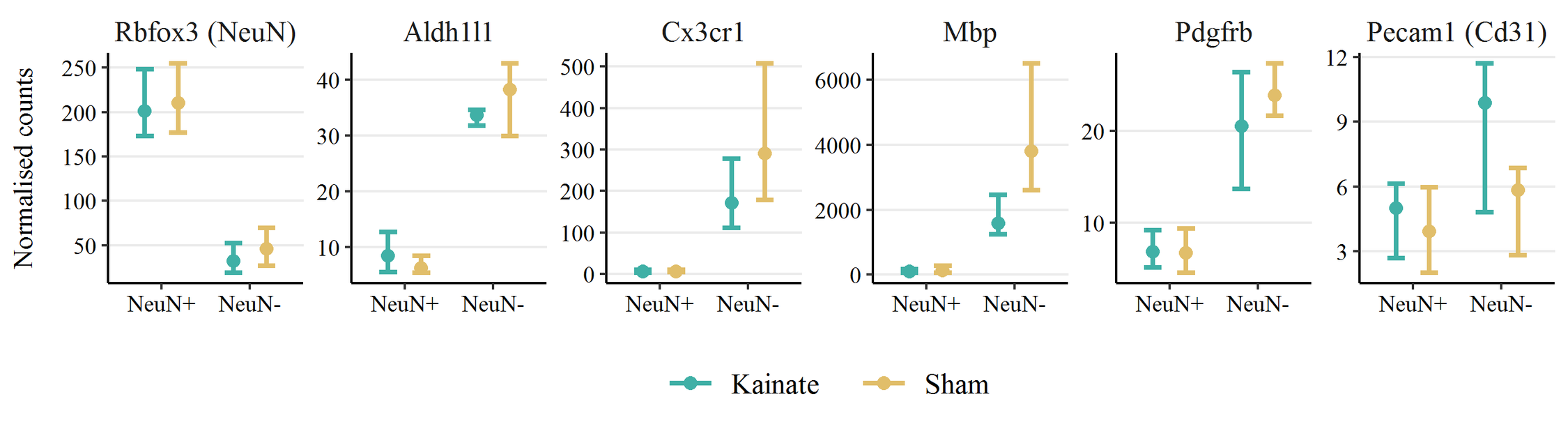

Supplement: S6 Fig — Neurons: RBFOX3 (= NeuN), Astrocytes: ALDH1L1, Microglia: CX3CR1, Oligodendrocytes: MBP, Pericytes: PDGFRB, Endothelial cells: PECAM1; Expression in the NeuN+ and NeuN- fraction on the left and right side of each graph. (TIF) [file pone.0226575.s008.tif]

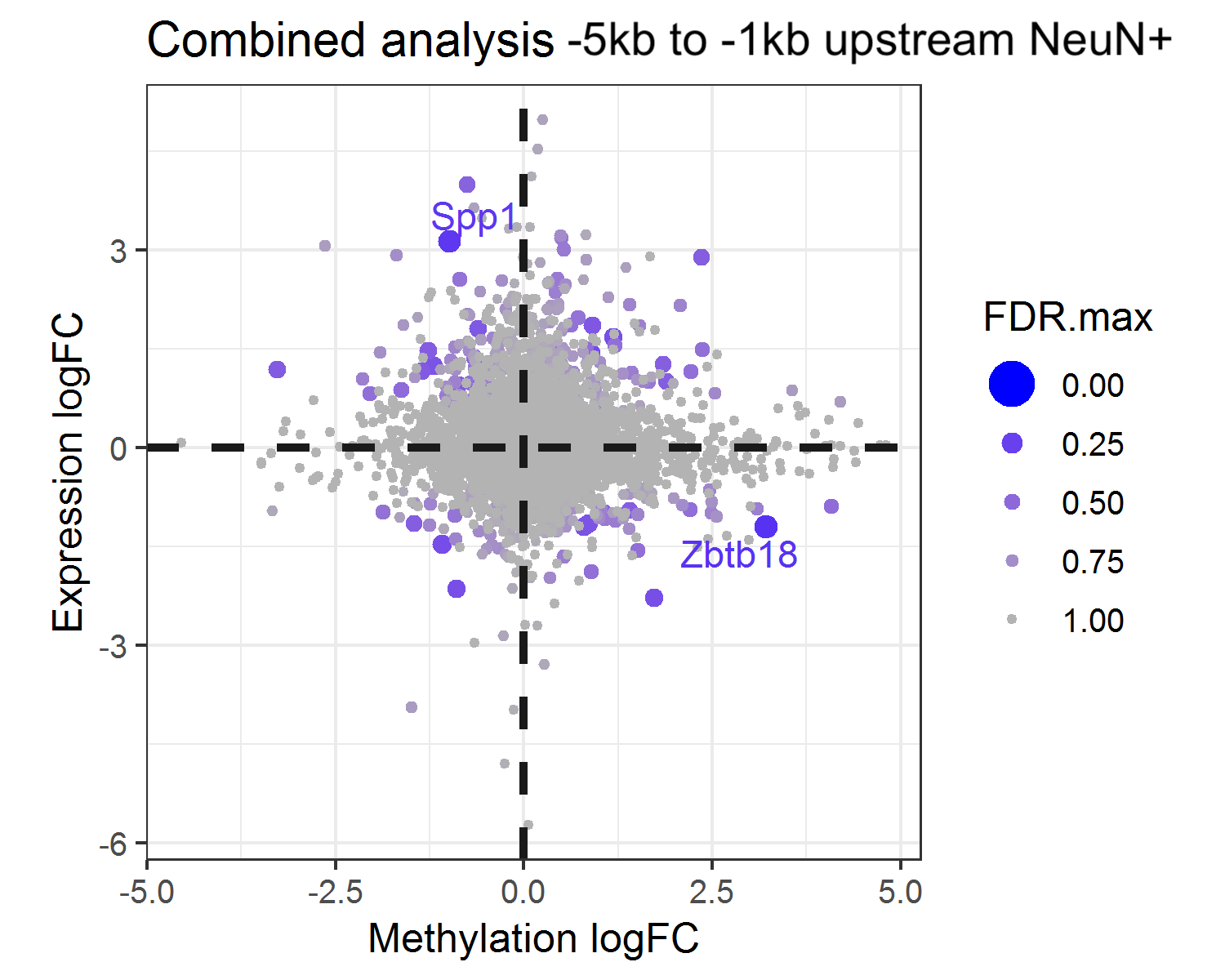

Supplement: S7 Fig — Visualization of DM and DGE (FDR 0.25) for neurons (upstream). Genes associated with significantly altered DMR and DGE are indicated in the figure. (TIF) [file pone.0226575.s009.tif]

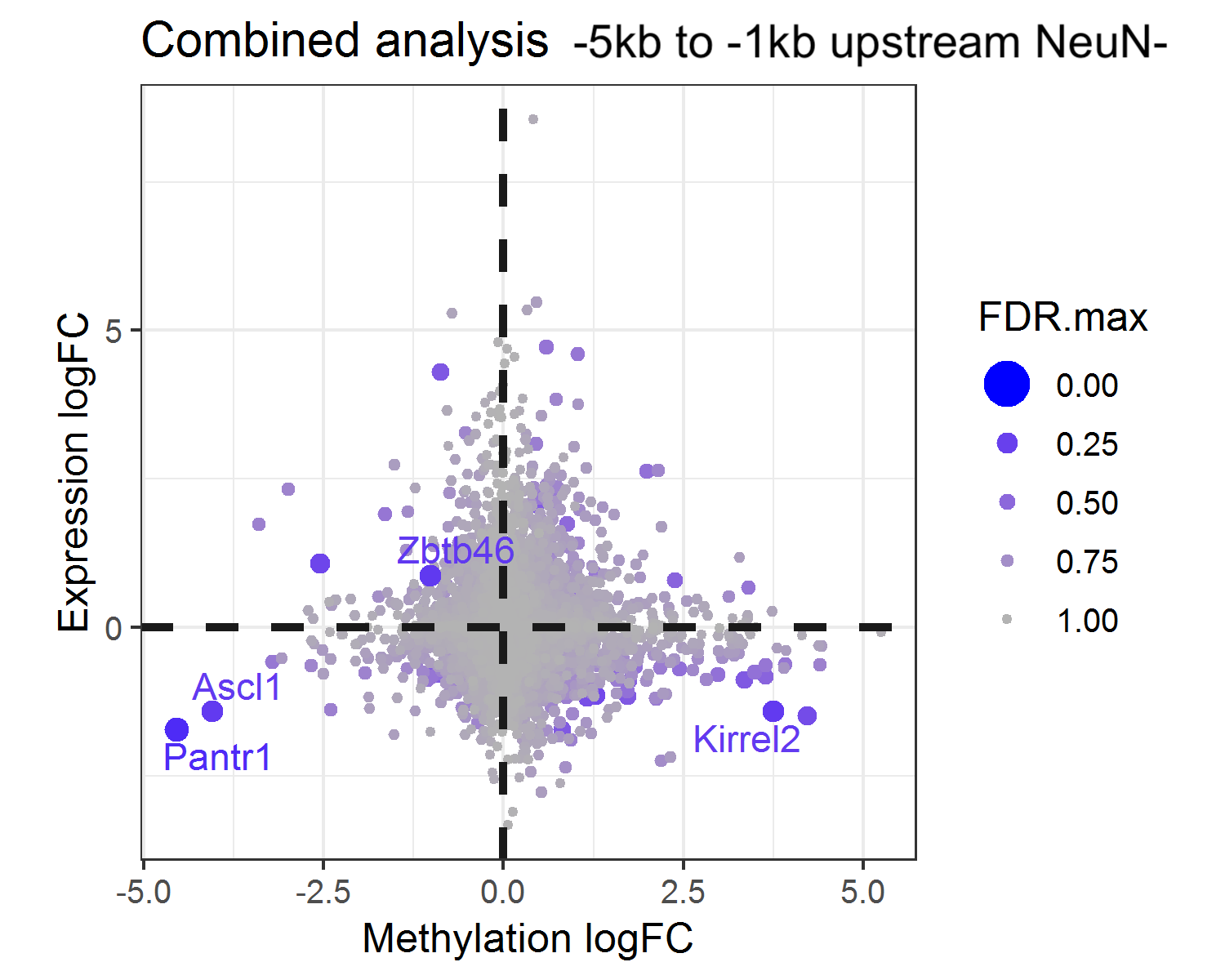

Supplement: S8 Fig — Visualization of DM and DGE (FDR 0.25) for glia (upstream). Genes associated with significantly altered DMR and DGE are indicated in the figure. (TIF) [file pone.0226575.s010.tif]

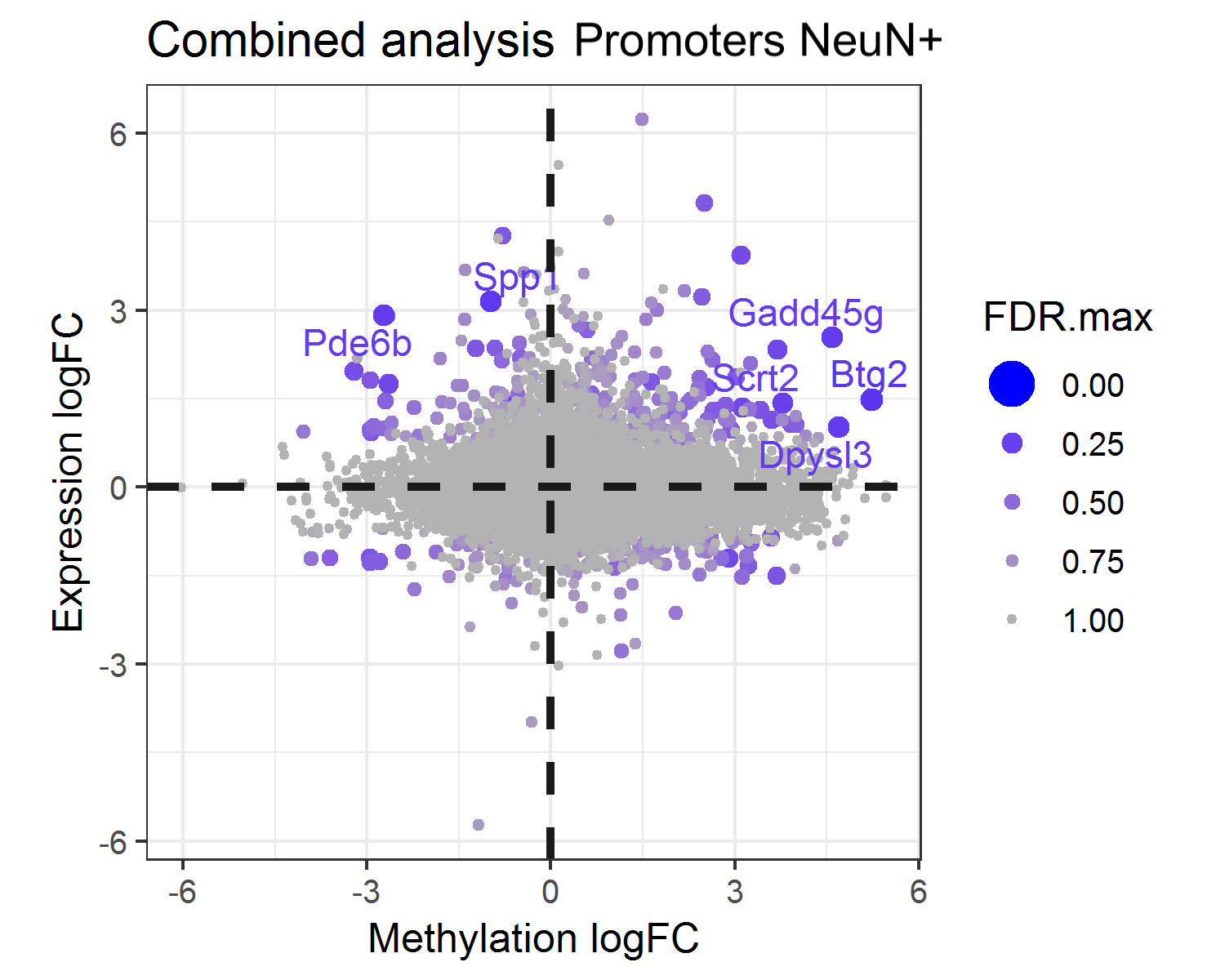

Supplement: S9 Fig — Visualization of DM and DGE (FDR 0.25) for neurons (promoters). Genes associated with significantly altered DMR and DGE are indicated in the figure. (TIF) [file pone.0226575.s011.tif]

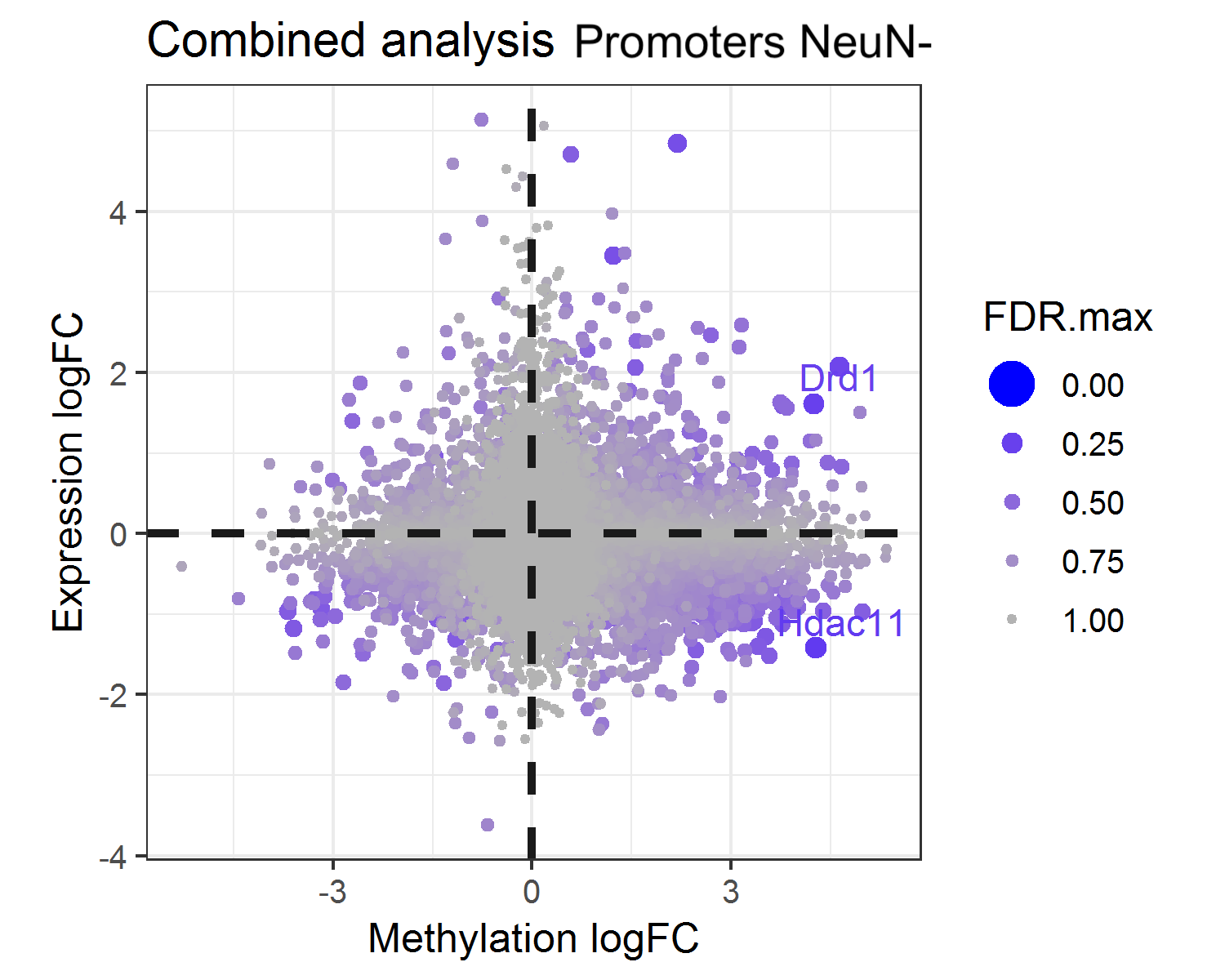

Supplement: S10 Fig — Visualization of DM and DGE (FDR 0.25) for glia (promoters). Genes associated with significantly altered DMR and DGE are indicated in the figure. (TIF) [file pone.0226575.s012.tif]

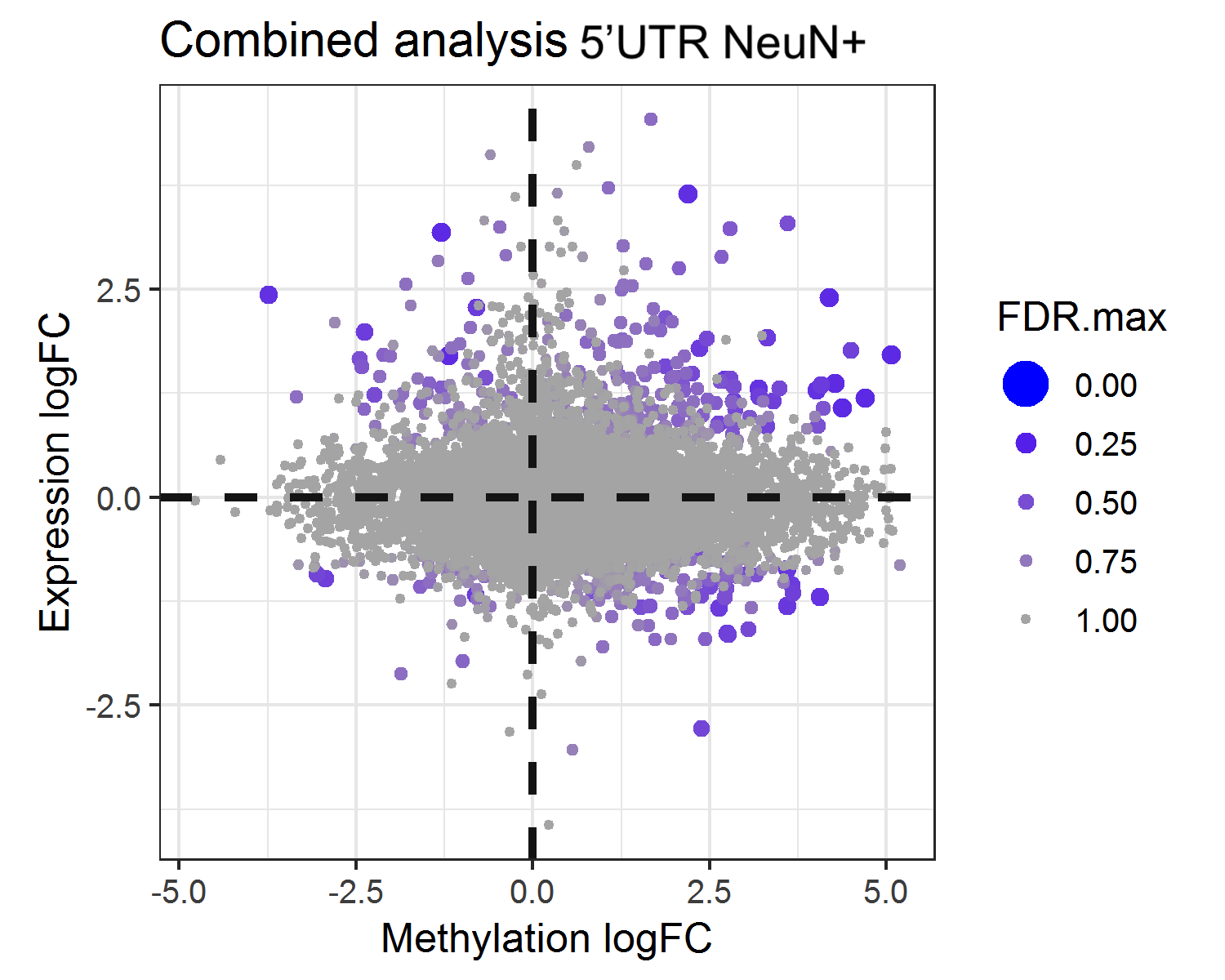

Supplement: S11 Fig — Visualization of DM and DGE (FDR 0.25) for neurons (UTR5). Genes associated with significantly altered DMR and DGE are indicated in the figure. (TIF) [file pone.0226575.s013.tif]

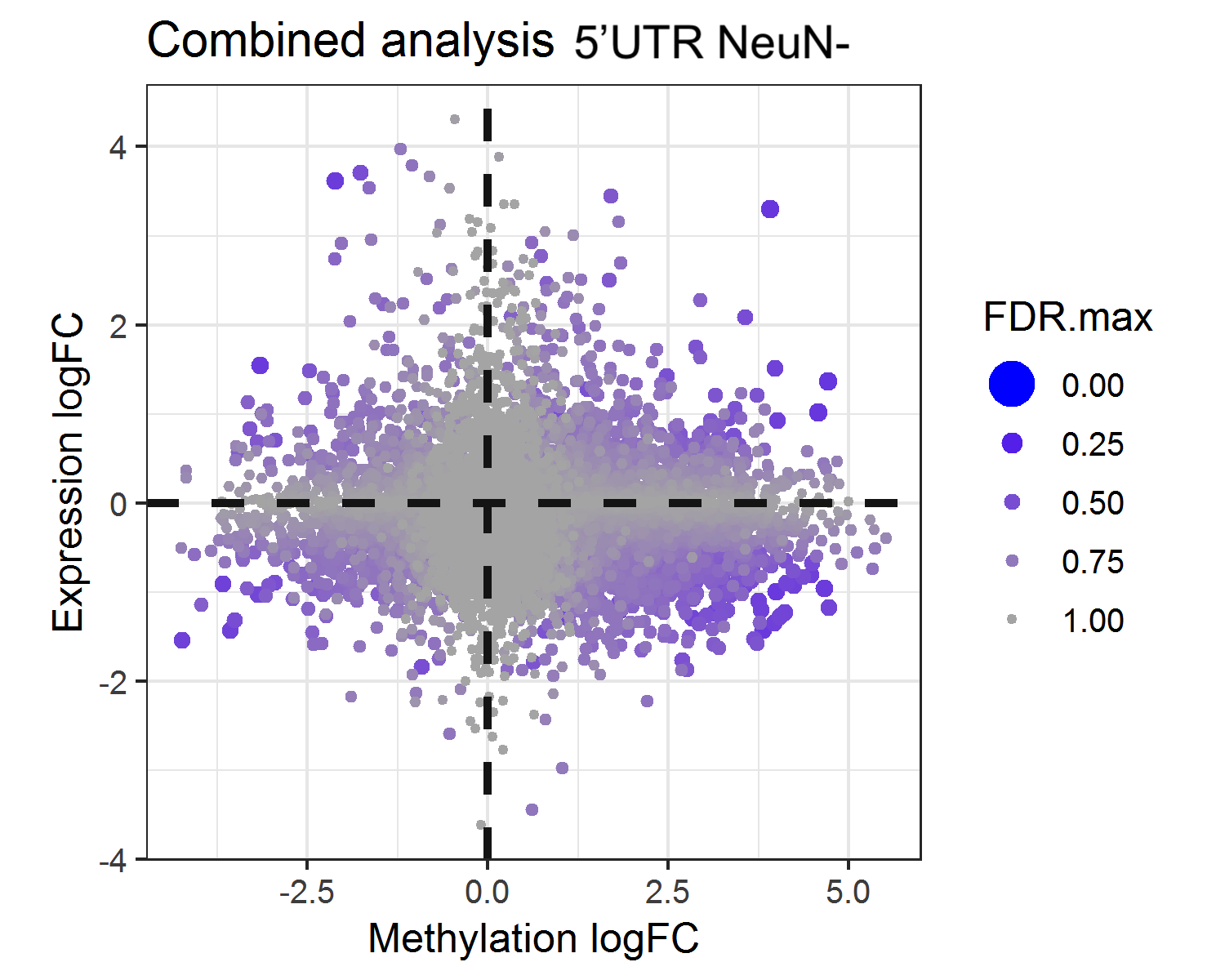

Supplement: S12 Fig — Visualization of DM and DGE (FDR 0.25) for glia (UTR5). Genes associated with significantly altered DMR and DGE are indicated in the figure. (TIF) [file pone.0226575.s014.tif]

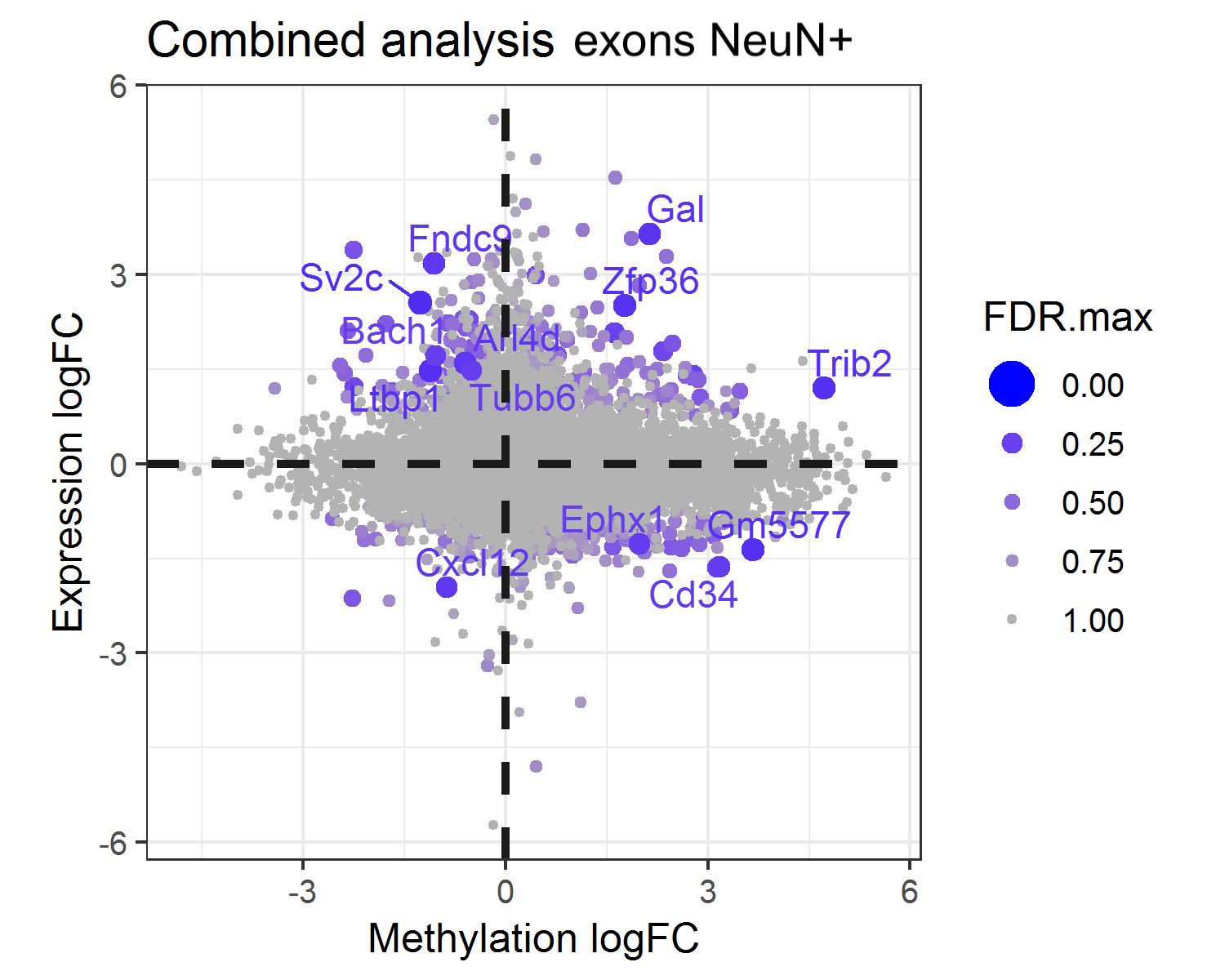

Supplement: S13 Fig — Visualization of DM and DGE (FDR 0.25) for neurons (exon). Genes associated with significantly altered DMR and DGE are indicated in the figure. (TIF) [file pone.0226575.s015.tif]

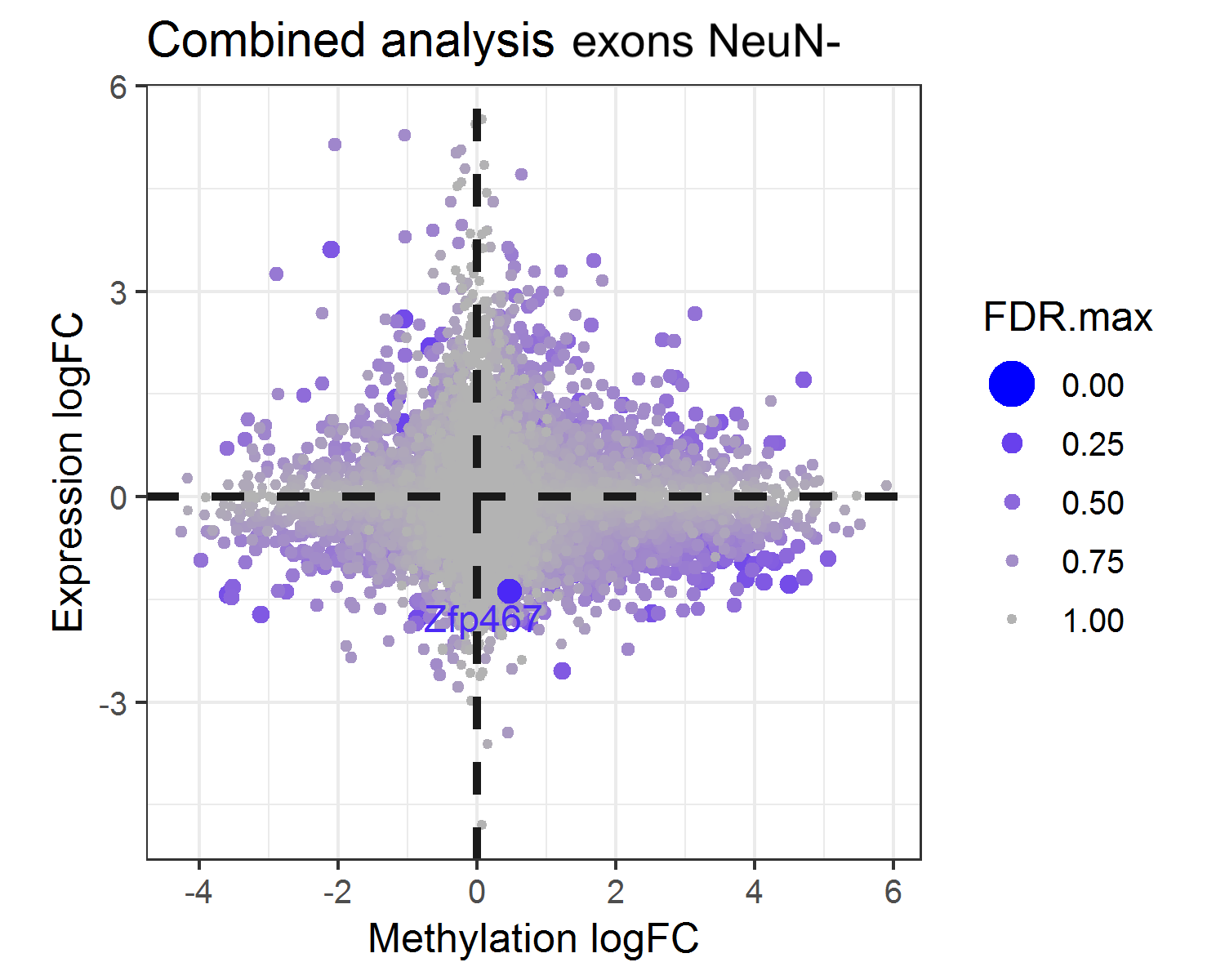

Supplement: S14 Fig — Visualization of DM and DGE (FDR 0.25) for glia (exon). Genes associated with significantly altered DMR and DGE are indicated in the figure. (TIF) [file pone.0226575.s016.tif]

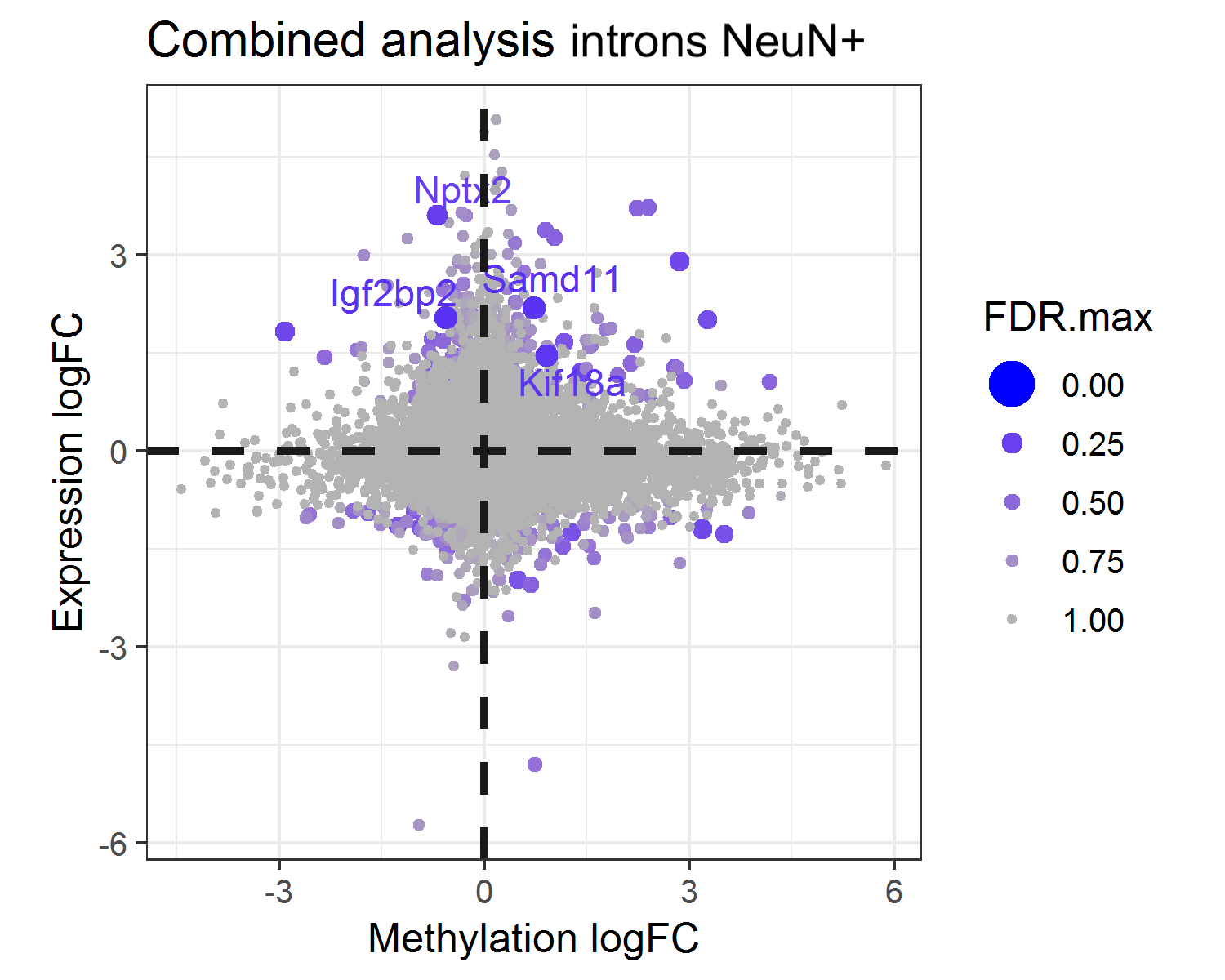

Supplement: S15 Fig — Visualization of DM and DGE (FDR 0.25) for neurons (intron). Genes associated with significantly altered DMR and DGE are indicated in the figure. (TIF) [file pone.0226575.s017.tif]

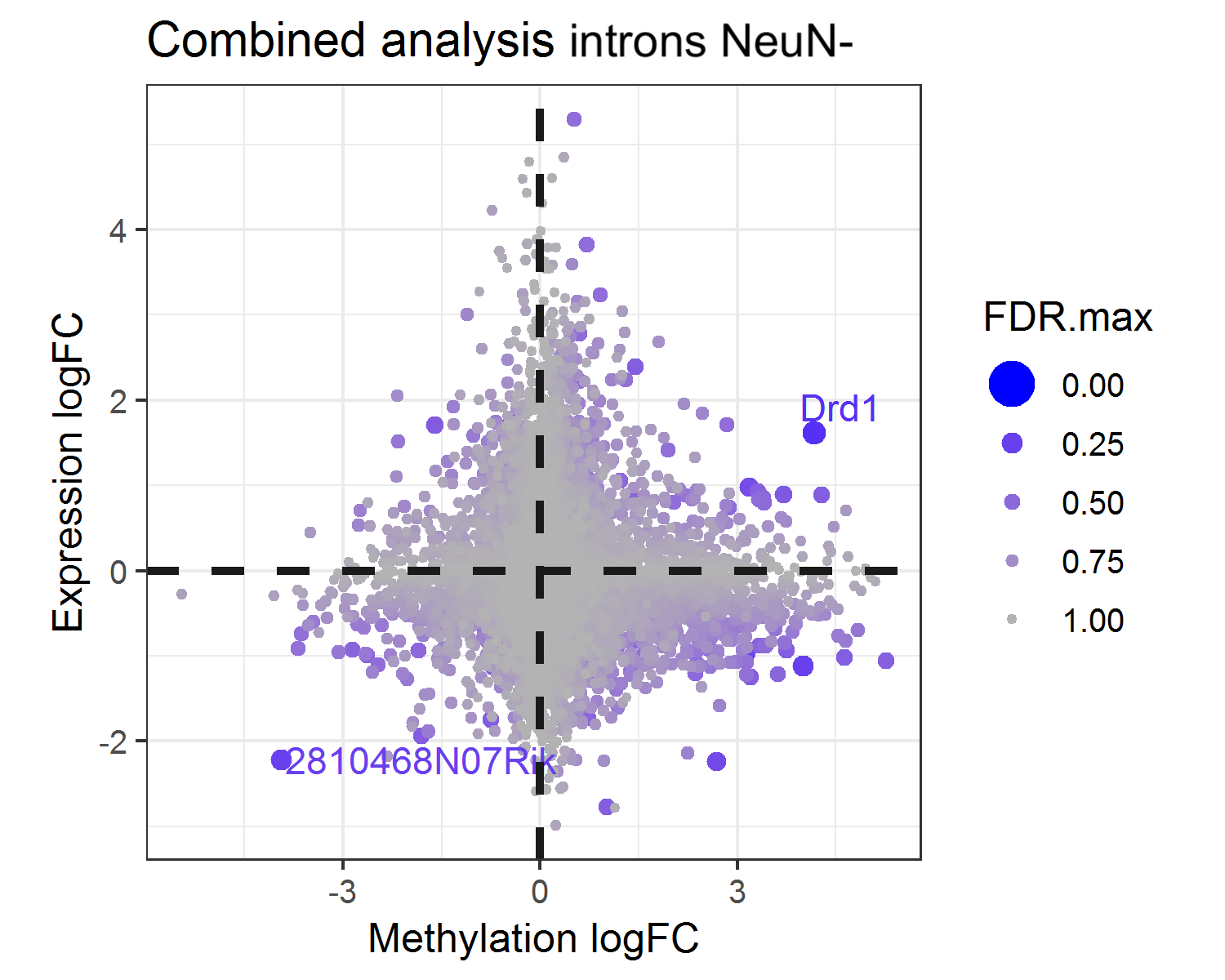

Supplement: S16 Fig — Visualization of DM and DGE (FDR 0.25) for glia (intron). Genes associated with significantly altered DMR and DGE are indicated in the figure. (TIF) [file pone.0226575.s018.tif]

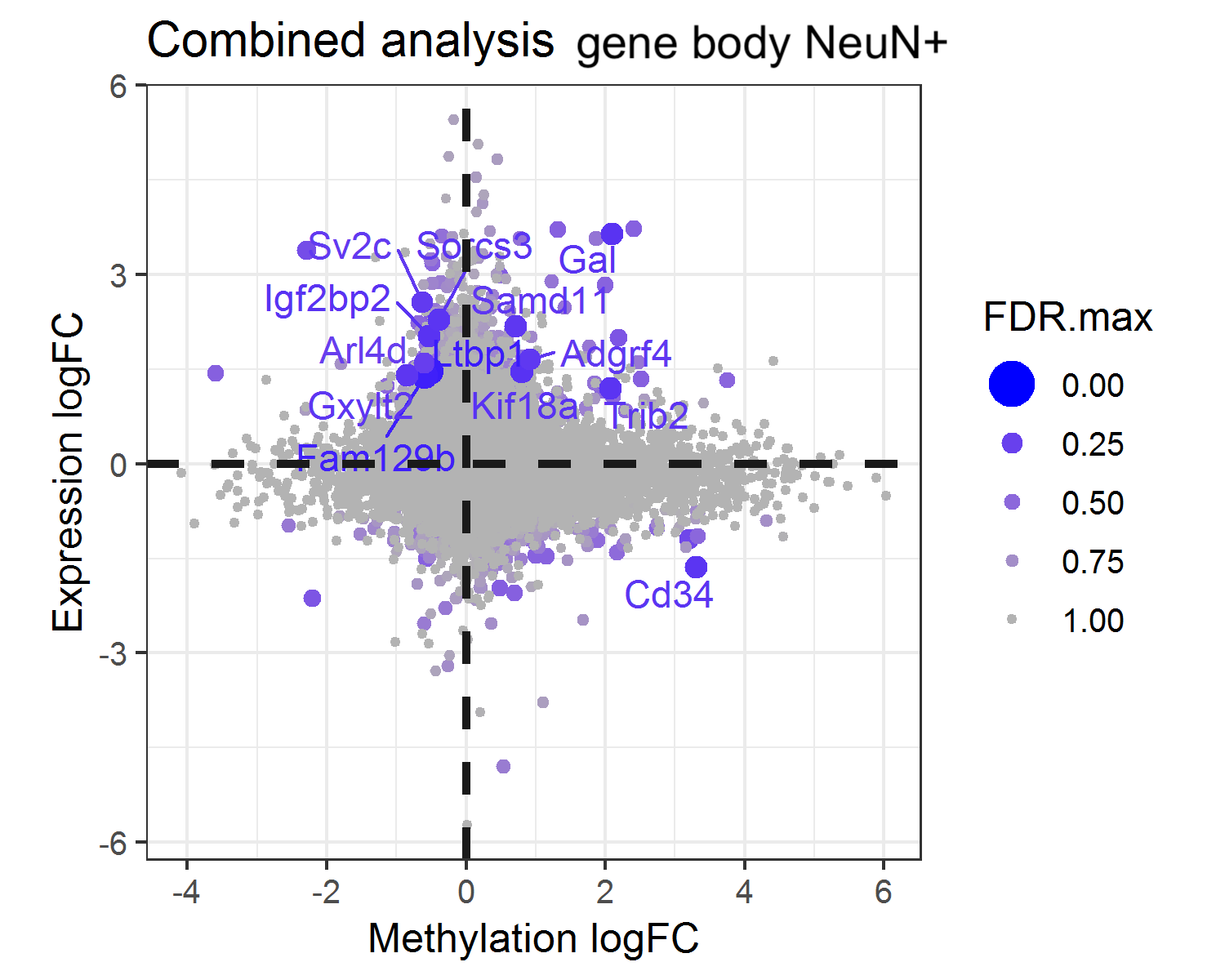

Supplement: S17 Fig — Visualization of DM and DGE (FDR 0.25) for neurons (gene body). Genes associated with significantly altered DMR and DGE are indicated in the figure. (TIF) [file pone.0226575.s019.tif]

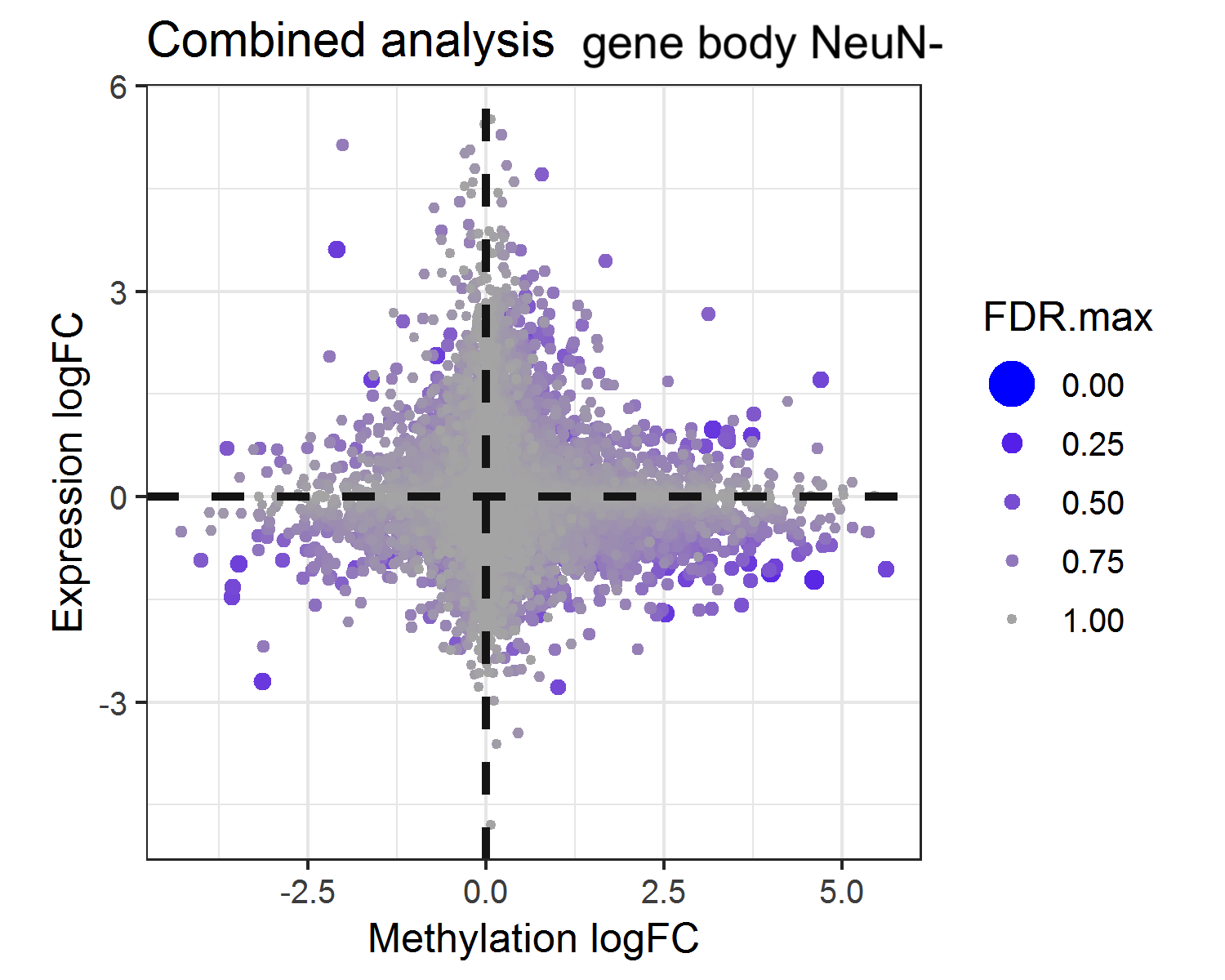

Supplement: S18 Fig — Visualization of DM and DGE (FDR 0.25) for glia (gene body). Genes associated with significantly altered DMR and DGE are indicated in the figure. (TIF) [file pone.0226575.s020.tif]

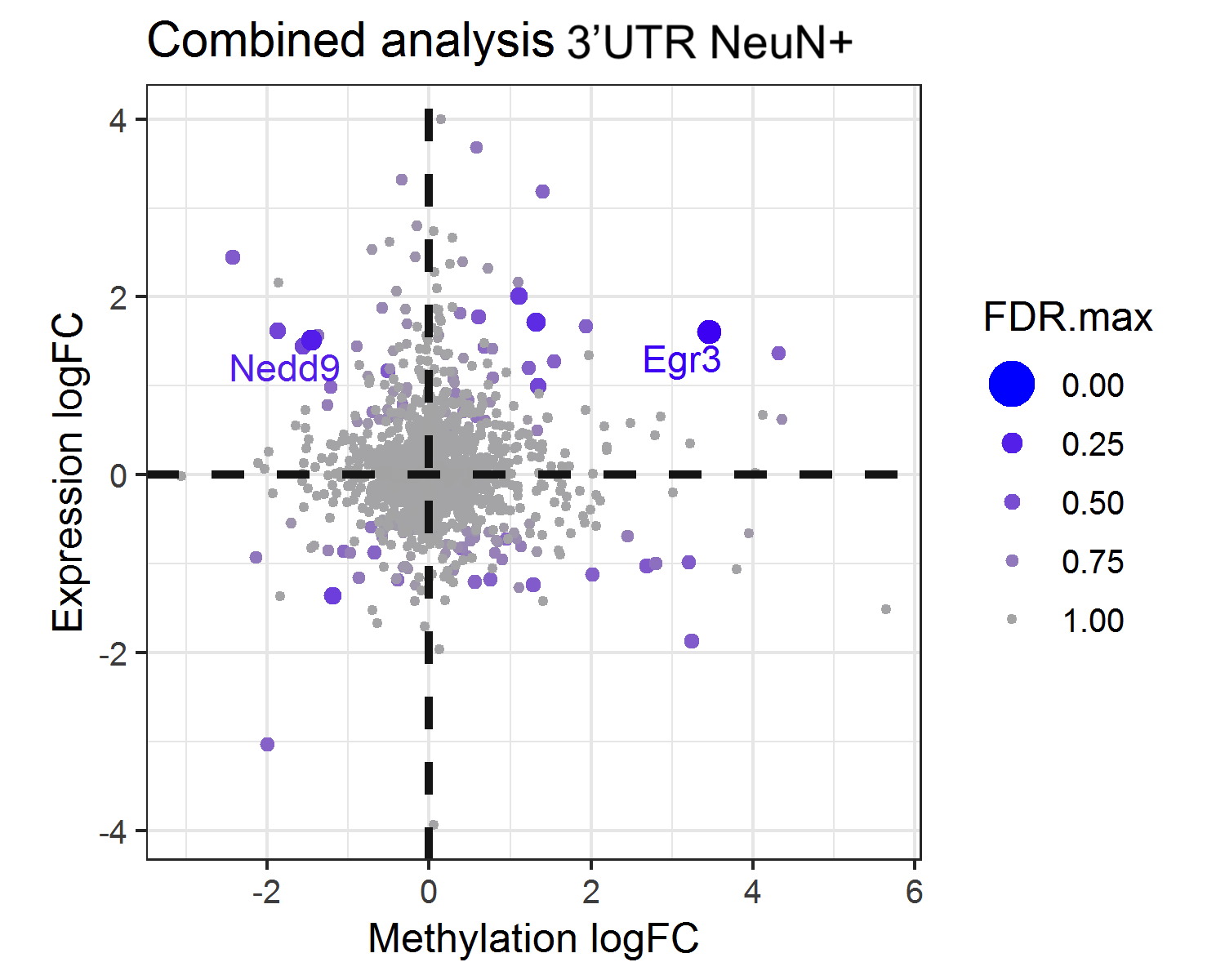

Supplement: S19 Fig — Visualization of DM and DGE (FDR 0.25) for neurons (UTR3). Genes associated with significantly altered DMR and DGE are indicated in the figure. (TIF) [file pone.0226575.s021.tif]

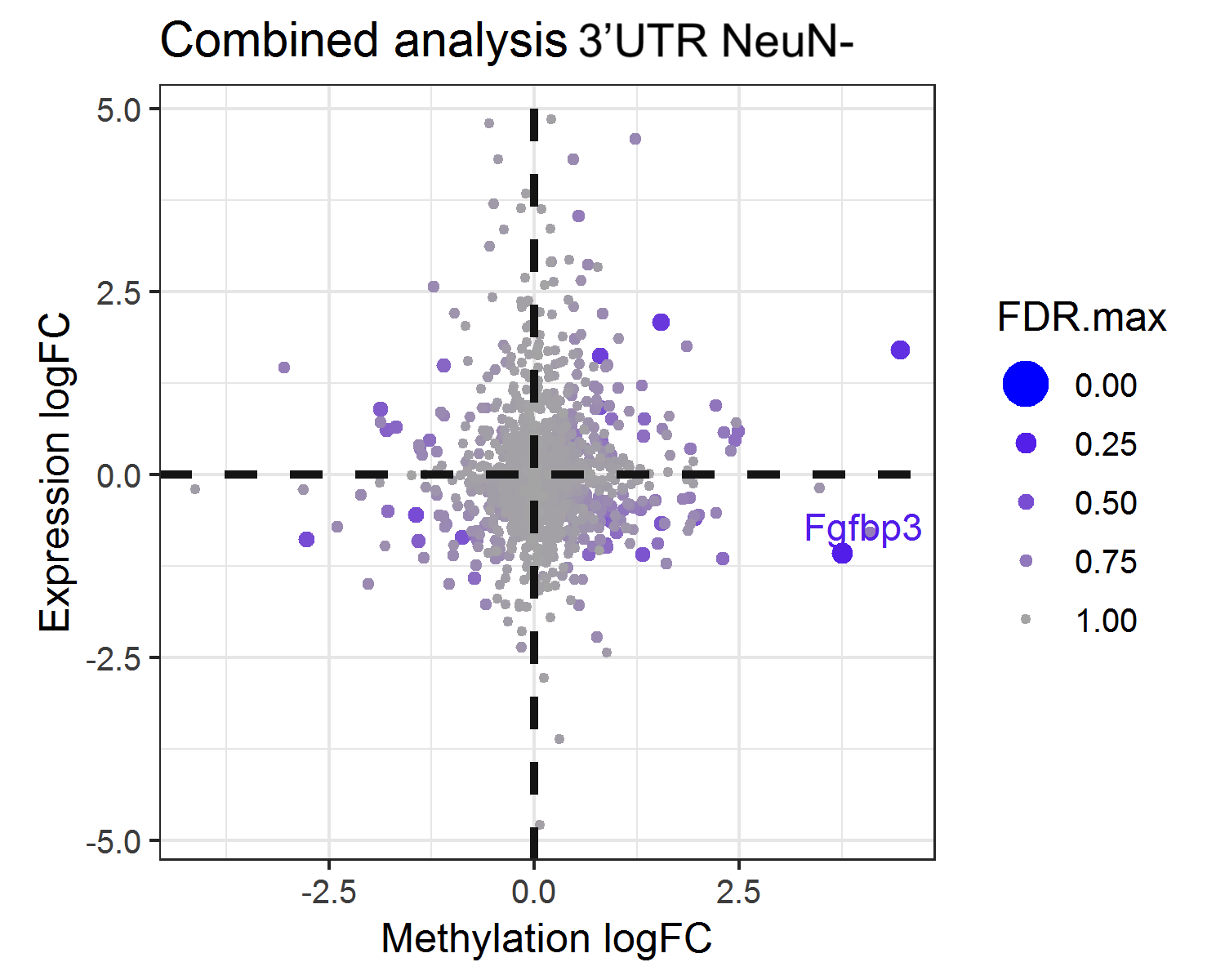

Supplement: S20 Fig — Visualization of DM and DGE (FDR 0.25) for glia (UTR3). Genes associated with significantly altered DMR and DGE are indicated in the figure. (TIF) [file pone.0226575.s022.tif]
