# Supplementary material for: Neuronal and glial DNA methylation and gene expression changes in early epileptogenesis
Source: PLoS One. 2019 Dec 30;14(12):e0226575. doi: 10.1371/journal.pone.0226575 (PMC6936816; doi:10.1371/journal.pone.0226575)
Supplement: Attachment — Submitted filename: Response to Reviewers.docx [file pone.0226575.s024.docx]

|  |  |  |
| --- | --- | --- |
|  | **Kjell Heuser** *M.D., Ph.D.*  Senior Consultant Neurologist and  Senior Researcher  Epilepsy Research Group of Oslo Department of Neurology  Oslo University Hospital  P.O. Box 4950 Nydalen  0424 Oslo, Norway  m. +47 92414568  kjell.heuser@ous-hf.no | |

Manuscript number: PONE-D-19-20265

Title: Neuronal and glial DNA methylation and gene expression changes in early epileptogenesis

Dear Editor Professor Biagini,

We thank you for your feedback regarding our paper:

- Indicate how animals were killed.

- Provide a section describing statistics in methods.

- Check tables for values' unit (thousands).

All three points have been addressed and the manuscript adjusted accordingly.

We hope that you will find the new version suitable for publication in PLOS ONE.

With kind regards,

Kjell Heuser
